# Disruption of the odorant coreceptor *Orco* impairs foraging and host finding behaviors in the New World screwworm fly

**DOI:** 10.1038/s41598-021-90649-x

**Published:** 2021-05-31

**Authors:** Daniel F. Paulo, Ana C. M. Junqueira, Alex P. Arp, André S. Vieira, Jorge Ceballos, Steven R. Skoda, Adalberto A. Pérez-de-León, Agustin Sagel, William O. McMillan, Maxwell J. Scott, Carolina Concha, Ana M. L. Azeredo-Espin

**Affiliations:** 1 Department of Genetics, Evolution, Microbiology and Immunology, Institute of Biology, University of Campinas (UNICAMP), Campinas, SP 13083-875 Brazil; 2 Department of Genetics, Institute of Biology, Federal University of Rio de Janeiro (UFRJ), Rio de Janeiro, RJ 21941-902 Brazil; 3 USDA-ARS, Knipling-Bushland U.S. Livestock Insects Research Laboratory and Veterinary Pest Genomics Center, Kerrville, TX 78028 USA; 4 Department of Structural and Functional Biology, Institute of Biology, University of Campinas (UNICAMP), Campinas, SP 13083-862 Brazil; 5 Microscopy Laboratory, Smithsonian Tropical Research Institute (STRI), Tupper Building, Panama city, 0843-03092 Panama; 6 USDA-ARS, Knipling-Bushland U.S. Livestock Insects Research Laboratory and Veterinary Pest Genomics Center, Screwworm Research Site, Pacora, Panama; 7 USDA-ARS, San Joaquin Valley Agricultural Sciences Center, Parlier, CA 93648 USA; 8 Laboratory of Ecological and Evolutionary Genomics, Smithsonian Tropical Research Institute (STRI), Gamboa, Panama; 9 Department of Entomology and Plant Pathology, North Carolina State University, Campus Box 7613, Raleigh, NC 27695 USA

**Keywords:** Agricultural genetics, Functional genomics

## Abstract

The evolution of obligate ectoparasitism in blowflies (Diptera: Calliphoridae) has intrigued scientists for over a century, and surprisingly, the genetics underlying this lifestyle remain largely unknown. Blowflies use odors to locate food and oviposition sites; therefore, olfaction might have played a central role in niche specialization within the group. In insects, the coreceptor *Orco* is a required partner for all odorant receptors (ORs), a major gene family involved in olfactory-evoked behaviors. Hence, we characterized the *Orco* gene in the New World screwworm, *Cochliomyia hominivorax*, a blowfly that is an obligate ectoparasite of warm-blooded animals. In contrast, most of the closely related blowflies are scavengers that lay their eggs on dead animals. We show that the screwworm *Orco* orthologue (*ChomOrco*) is highly conserved within Diptera, showing signals of strong purifying selection. Expression of *ChomOrco* is broadly detectable in chemosensory appendages, and is related to morphological, developmental, and behavioral aspects of the screwworm biology. We used CRISPR/Cas9 to disrupt *ChomOrco* and evaluate the consequences of losing the OR function on screwworm behavior. In two-choice assays, *Orco* mutants displayed an impaired response to floral-like and animal host-associated odors, suggesting that OR-mediated olfaction is involved in foraging and host-seeking behaviors in *C*. *hominivorax*. These results broaden our understanding of the chemoreception basis of niche occupancy by blowflies.

## Introduction

Identifying the genomic changes underlying adaptation in living organisms is a fundamental goal of modern evolutionary biology. In the context of insect evolution, the emergence of parasitic lineages offers a unique opportunity to investigate the genetic basis of adaptive traits driving species to occupy novel ecological niches. As noted by Grimaldi and Engel (2005), “*Calyptratae flies have redefined the ‘art’ of vertebrate parasitism, particularly the Oestroidea* […]^1^”, a superfamily that includes blowflies, botflies, fleshflies and relatives. Within Oestroidea, the family Calliphoridae is of particular interest due to its synanthropic habits. Commonly known as blowflies, the members of this family are frequently found foraging on plant inflorescence, where they feed on nectar^2^, as well as breeding on decaying organic matter and carcasses^3^. Therefore, blowflies have important roles in nature as pollinators and as recyclers of organic waste. In addition, they also have a remarkable importance in forensics^3^, veterinary^4^, and medical sciences^5,6^. Although better known for these necro-saprophagous flies, the group spans an even greater diversity of life-history strategies, including many forms of parasitism (examples are given by^7^). In particular, the evolution of obligate ectoparasitism is one of the most outstanding events within Calliphoridae, which has intrigued entomologists and evolutionary biologists over decades^7–11^. Estimations of the divergence timescale support that this lifestyle arose recently and independently at least twice after the explosive radiation of blowflies about 22 million years (Ma) ago, having a free-living scavenger ancestor^7,10,12,13^. The diversification of grazing mammals during this period is thought to have created a favorable landscape for their rapid speciation^10^, which might have been followed by intense competition for ephemeral carrion resources. This harsh condition may have initially favored opportunistic blowflies attracted by decaying flesh in the surface of animal’s wounds to oviposit, ultimately leading to the evolution of obligate ectoparasite lineages^14^. However, while these studies have addressed the likely origins of obligate ectoparasitism in blowflies, there have been relatively few attempts at understanding the genetic basis underlying this lifestyle.

Here, we hypothesized that olfactory chemoreception may have played a critical role in the adaptive transition from a necro-saprophagous to an obligate ectoparasitic habit in blowflies. Olfaction is a core chemosensory process in sensory perception, and divergences in olfaction-related genes are known to contribute to premating isolation, speciation and niche adaptation in insects^15,16^. We adopted the New World screwworm, *Cochliomyia hominivorax* (Coquerel 1858), as our research model. The screwworm is the sole obligate ectoparasite among the *Cochliomyia* genus, which includes four endemic species to the Americas, in addition to nearly all of the closely related blowflies, which are primarily carrion feeders^10,17^. Adult screwworms feed on flower nectar while their larvae feed on the live tissues of animals. Gravid female screwworms rely on odors emitted from wounded warm-blooded vertebrates to find suitable hosts for oviposition^4,18,19^, and lay their eggs on the dried margins of wounds and bodily orifices of their selected animal hosts. After hatching, the larvae infest and consume the animal living tissues to complete their development. These traumatic infestations are known as myiasis, which can lead to death if untreated^5^. The devastating effects of this species on wildlife and livestock encouraged a decades-long eradication campaign, using the sterile insect technique (SIT), that successfully eliminated screwworms from North and Central America^20^. At present, millions of radiated-sterile screwworms are released daily along the Panama‐Colombia border. This barrier zone prevents and counter-strike *C*. *hominivorax* outbreaks, as recently seen in the United States^21^, from regions where the species remains endemic. Meanwhile, screwworms remain a serious problem for animal health in South America^5,22^. Because of its well-established phylogenetic status, its consequences as a destructive invasive pest, and the increasing availability of genomic resources^23,24^, *C*. *hominivorax* represents a promising model to address the genetic basis of niche occupancy in blowflies.

In insects, the sense of odors in complex environments is mediated by several chemosensory genes expressed in porous sensilla attached to olfactory organs, including the antennae and maxillary palps^25^. Inside the sensilla, odors are solubilized and shuttled through the inner lymph to receptor sites present in the olfactory sensory neurons (OSNs). Two distinct receptor families, named ionotropic receptor (IR) and odorant receptor (OR), are responsible for recognizing the intercepted signals, resulting in the activation of OSNs and a cascade of neural events leading to a multitude of behavioral responses^16,25–28^. Although the OR and IR gene families include a large number of members (for instance, 60 and 66 genes in *Drosophila melanogaster*, respectively^29^) the proper function of all divergent ORs is dependent on a common odorant receptor coreceptor, named *Orco*^27,30,31^. A failure to encode *Orco* results in abnormal behaviors driven by OR-mediated olfaction, while maintaining other chemosensory pathways intact^32–37^. In this context, an *Orco* knockout strain of *C*. *hominivorax* would provide a simple system to rapidly differentiate olfactory behaviors mediated by the OR and IR families in this species. In this study, we isolated the *Orco* orthologue of *C*. *hominivorax* (named *ChomOrco*), characterized its sequence in a phylogenetic context, and assessed its developmental and tissue expression patterns. We next expanded our previous CRISPR/Cas9 genome editing protocols^23^ to develop a germline *Orco* null strain, and evaluated the contribution of OR-mediated olfaction in foraging and host-seeking behaviors in *C*. *hominivorax*. The data presented here provides new functional evidence on the chemoreception basis of ecological specialization in the screwworm fly.

## Results

### The screwworm *Orco* orthologue is highly conserved within Diptera

**Figure 1:**
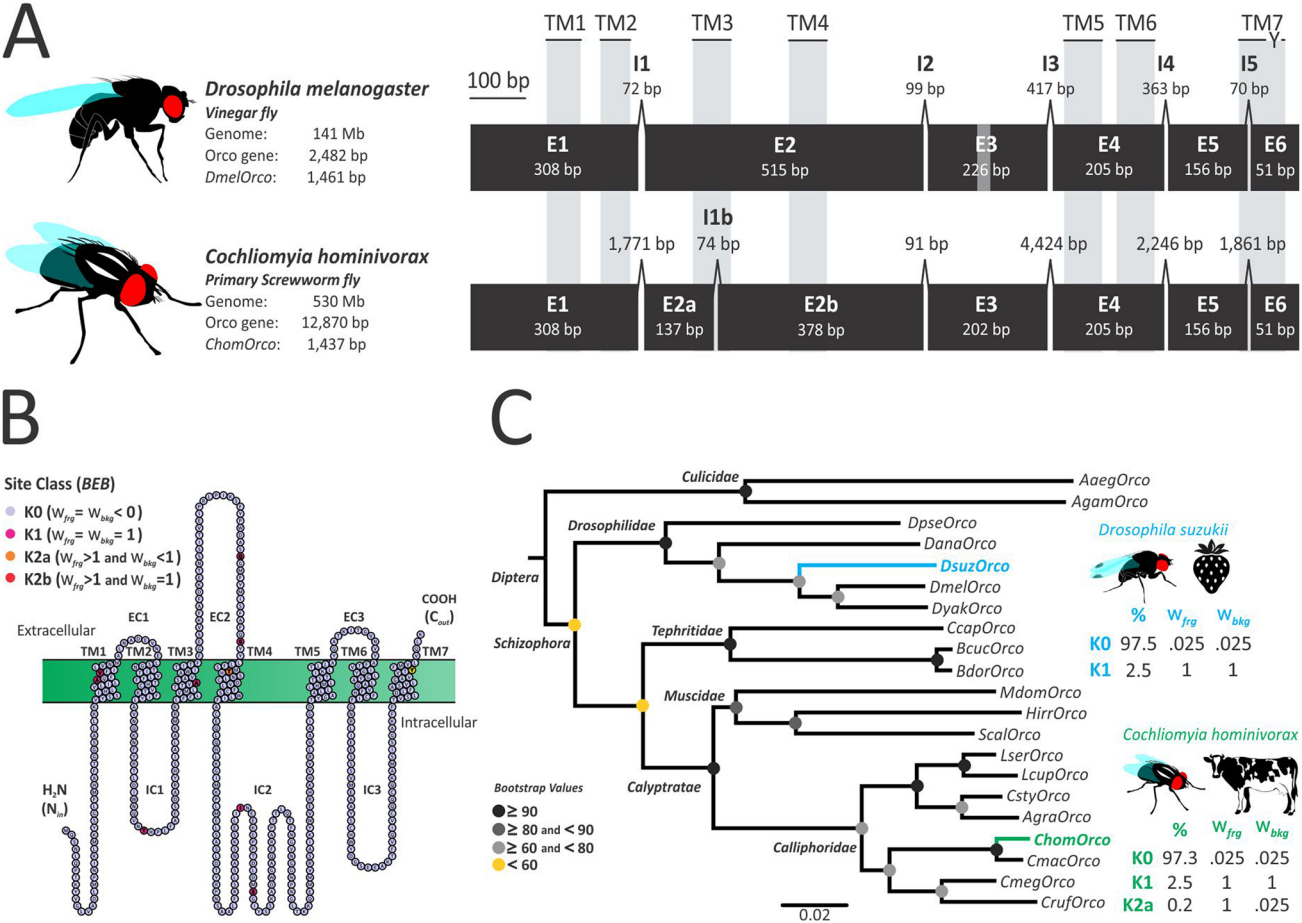
Screwworm *Orco* orthologue is highly conserved within diptera. (**A**) Comparison between the *Orco* genomic organization in *D*. *melanogaster* (*DmelOrco*) and *C*. *hominivorax* (*ChomOrco*). Exons are represented by numbered black boxes (E1-E6) and introns as connecting lines (I1-I5). The seven transmembrane domains (TM1-TM7), conserved tyrosine residue at TM7 (Y), and nucleotide length variation at E3 (gray strip) are also represented (dipteran’s *Orco* sequence alignment is shown in Supplementary Fig. S1). (**B**) Predicted protein topology of *ChomOrco* displaying significant characterized sites by Bayes Empirical Bayes (BEB). (**C**) Maximum Likelihood (ML) reconstruction of relationships between dipteran *Orco* sequences (species and accessions used can be found in Supplementary Table S3). Bootstrap support values, estimated from 500 non-parametric replications, are shown at their respective nodes. Normalized non-synonymous (d_N_) to synonymous (d_S_) substitution rates (ω) were estimated to test the branches leading to *C*. *hominivorax* (in green) and *D*. *suzukii* (in blue) species for events of episodic diversification. The number of sites estimated to be evolving under purifying (K0), relaxed (K1), and positive (K2a or K2b) selection is shown for these lineages (foreground branch; ω_frg_) in relation to the rest of the tree (background branch; ω_bkg_).

Classic-RACE was used to isolate the full-length *Orco* transcript sequence of *C*. *hominivorax*, which consists of 1437 base pairs (bp) encoding a 478 amino-acid (aa) peptide sequence. The *ChomOrco* transcript shares 73 and 92% of nucleotide identity with *Orco* sequences from *D*. *melanogaster* and the closely related Oriental latrine blowfly, *Chrysomya megacephala*, respectively. In addition, the *ChomOrco* coding sequence shows an extremely high aa identity with all dipterans investigated in this study (mean ± SD: 92 ± 5.5%; Supplementary Table S1). Based on its transcript sequence, we next used a combination of bioinformatics genome-wide analysis and long-range PCR sequencing to isolate the complete 12,870 bp genomic region corresponding to the *Orco* gene in screwworm. Genomic organization of *ChomOrco* is characterized by the presence of seven exons, highly conserved among dipterans, separated by six introns (Fig. 1A and Supplementary Fig. S1). Differently from *D*. *melanogaster* (*DmelOrco*), the exon 2 of screwworm *Orco* is subdivided into two parts (referenced here as E2a and E2b) separated by a 74 bp intronic region (named I1b). We further investigated the presence of intron I1b in other species and found that this region is not unique to the *C*. *hominivorax* genome, but rather has an ancient origin of at least ca. 50 Ma within the Schizophora clade, presumably being shared by all Calyptratae flies (Supplementary Fig. S1). Membrane protein topology predictions indicated that *ChomOrco* contains seven transmembrane domains (TM), an inverted terminal membrane topology (N_in_-C_out_) and a conserved tyrosine residue at TM7 (Fig. 1B), all signatures of this atypical OR coreceptor^27,38^. Most conserved residues were found at TM6 and TM7, which is thought to be a region where ORs partially interact with *Orco*. As evidenced in other calyptrates^39^, *ChomOrco* is eight amino acids (^309^NGGGGNGL^316^) shorter than *Drosophila* at the intracellular loop 2 (IC2), which connects the TM4 and TM5, a region believed to be important for intracellular transport^27^. The long IC2, in comparison with the conventional ORs, is another *Orco* feature^31,40^ and this region appears to be a common place for *Orco* length variations in dipteran species (Supplementary Fig. S1).

Gene tree inferences recovered *ChomOrco* in the Calliphoridae clade sharing a common node with *Orco* sequences of Muscidae species. The clade composed of Calliphoridae + Muscidae forms a sister-clade with Drosophilidae or Tephritidae families (Fig. 1C), consistently reflecting the phylogenetic relationships among the Schizophora clade^10^. We investigated the branch leading to *C*. *hominivorax* species for site-specific signatures of episodic diversification, as positive selection pressures are likely to affect only a few sites within a specific lineage. Results revealed that the selective pressures in the screwworm lineage do not differ from the background tree, presenting signals of a strong purifying regime (ω_K0_ = 0.025) in the majority of the corresponding aa sites (97.3%), while remaining sites showed evidence of relaxed constraint (ω_K1_ ∼ 1; Fig. 1B,C). Only a single site (^211^threonine, located inside TM4) exhibited some signal of positive selection in the *Cochliomyia* clade (ω_frg_ ≥ 1, while ω_bkg_ < 1), although it was not statistically significant (BEB score: 0.92). Thus, it’s likely that this site is rather experiencing a relaxation of selective constraints in this clade. The same evolutionary signatures were obtained when testing the branch leading to *Drosophila suzukii* lineage (*DsuzOrco*; Fig. 1C), illustrating that the *Orco* sequence conservation reflects its indispensable role in olfaction across taxa.

### Expression of *Orco* is conserved among blowflies and broadly detected in chemosensory-related tissues of *C. hominivorax* adults

**Figure 2:**
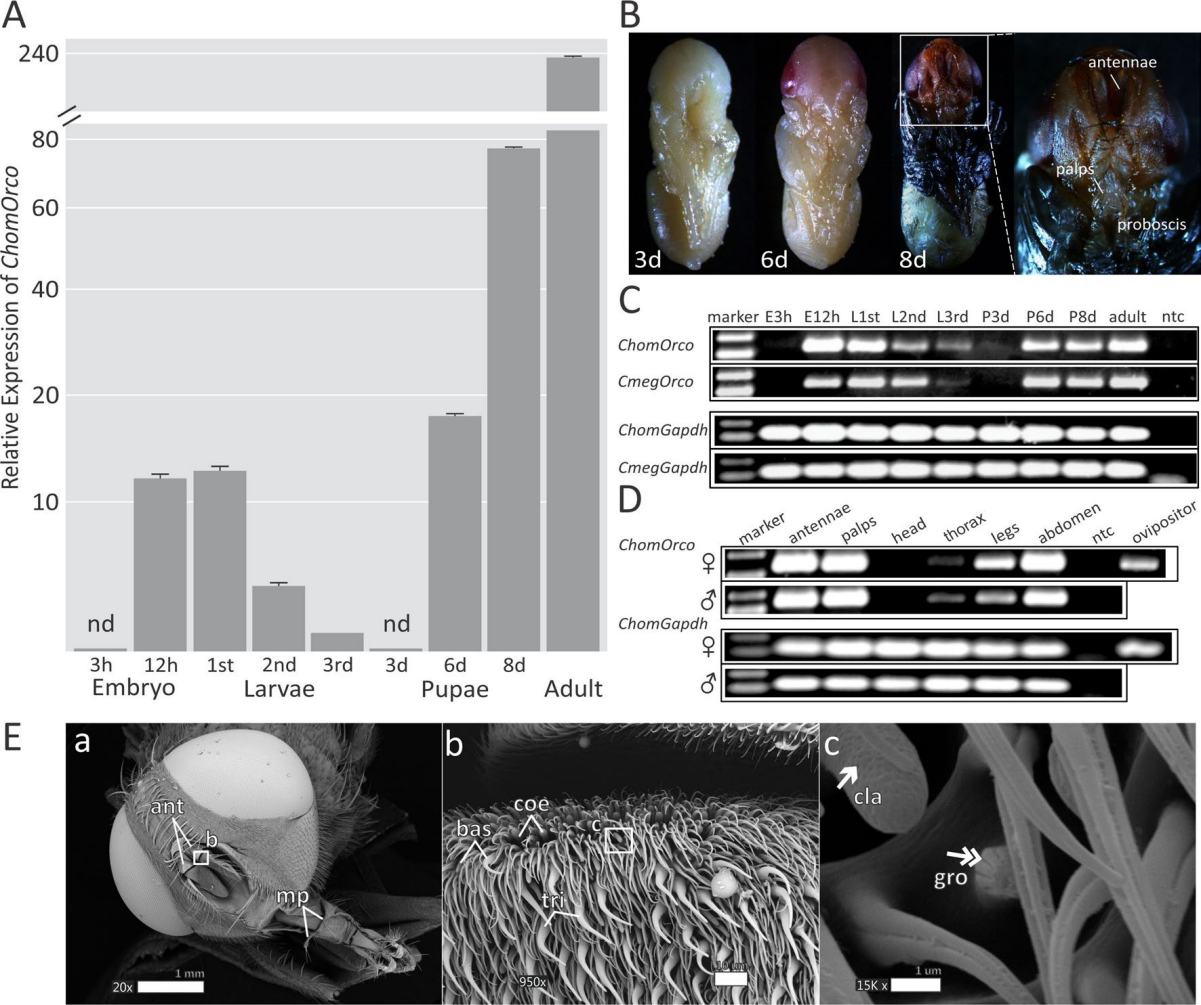
Expression of *Orco* is conserved among blowflies and broadly detected in chemosensory-related tissues of *C*. *hominivorax* adults. (**A**) Relative expression of *ChomOrco* during the screwworm development by qPCR. Measurements are given by the quantification of *ChomOrco* normalized to *GAPDH* using the 2^−ΔCt^ method and presented as fold-change relative to the third instar larvae using the 2^−ΔΔCt^ method. Data are represented as mean ± SD (*n* = 9). Cycle thresholds above 35 were considered non-detected (nd). (**B**) Intrapuparial development of *C*. *hominivorax* at three (3d), six (6d), and eight days (8d) after pupation. A closer view of the head region (white square) reveals the fully developed sensory structures of the adult form present in the late pupae stage. (**C**) Semi-quantitative comparison between developmental *Orco* expression in *C*. *hominivorax* (*Chom*) and *C*. *megacephala* (*Cmeg*) species by RT-PCR. (**D**) Detection of *ChomOrco* in adult screwworm female (♀) and male (♂) tissues. For all RT-PCR assays: Amplifications were made in replicates (*n* = 3), including no template controls (ntc), and *GAPDH* was amplified as an internal control. Cropped images (delineated by black lines) are from samples run on different gels. Full-length gels are displayed in Supplementary Fig. S2. (**E**) The main olfactory structures of *C*. *hominivorax* viewed under electron microscopy. (*panel a*) Screwworm female's head highlighting the antennae (ant), and the maxillary palps (mp). (*panel b)* A closer view of the proximal surface of the third antenna segment reveals a number of tricoide (tri), basiconic (bas), and two morphotypes of coelonic (coe) sensilla (*panel c*), named grooved (gro) and clavate (cla). These morphotypes are adorned with multi-wall pores (single arrows) and grooves (double arrows), which presumably facilitate the entrance of odor molecules into the antennae.

We investigated the relative expression of *Orco* during screwworm development by quantitative real-time PCR (qPCR). Results revealed that *Orco* transcription takes place during the final stages of embryogenesis (12 h after oviposition when eggs are incubated at 37 ± 2 °C), and its expression is maintained at similar levels during the first instar larvae stage (Fig. 2A). The *ChomOrco* expression decreases in the subsequent larval development, reaching the lowest levels in the third larval stage. The pupal stage is characterized by a gradual increase in *Orco* transcript abundance over time (3-to-8-days after pupation; Fig. 2A), reaching its highest level during the pre-imago development (8-days after pupation). Upon dissection (Fig. 2B; Supplementary Methods), it was possible to verify that the full development of the adult form and their main olfactory appendages occurs in this last pupal stage, which is synchronized with *ChomOrco* expression. In the adult stage, the *ChomOrco* transcription reaches its highest level. These observations are consistent with the idea that *Orco* expression follows the transition from a primordial larvae sensory system to more complex structures in adults^30^, also suggesting that olfaction plays a major role in the adult life stage of the screwworm fly. A similar developmental expression of *Orco* was observed in the blowfly *C*. *megacephala* (Fig. 2C) by semi-quantitative reverse transcription PCR (RT-PCR), suggesting that the regulation of *Orco* expression is conserved in closely related blowflies.

In insects, *Orco* is expressed in nearly all of the olfactory sensory neurons (OSNs^30^). Therefore, it was foreseen that *ChomOrco* would be mainly detected in olfactory-related tissues of *C*. *hominivorax* adults. Indeed, semi-quantitative amplifications revealed that *ChomOrco* is highly expressed in the main olfactory appendages of both sexes in the screwworm fly, including the antennae and the maxillary palps (Fig. 2D). Screwworm’s antennae are subdivided into three segments: the scape, pedicel and funiculus. The former accommodates a thin and plumose arista, as seen in Fig. 2E (panel *a*). Scanning electron microscopy (SEM; Supplementary Methods) of the female's head revealed that *C*. *hominivorax* funiculus is predominantly adorned by three classes of sensilla: coelonic, tricoide and basiconic (Fig. 2E; panel *b*). A close view of the proximal portion of this segment exposed a number of other two morphotypes of coeloconic sensilla, named grooved and clavate^41^, lying in deep bristle pits (Fig. 2E; panel *c*). These subclasses of sensilla are characterized by the presence of grooves and multiple wall-pores, which presumably allows the entry of molecules able to stimulate the chemoreception system located inside the antennae. Thus, the high level of *ChomOrco* transcription in the antennae is correlated to the morphology of this appendage. In addition to classic olfactory organs, a considerable abundance of *Orco* transcripts was also detected in legs and abdomen of both sexes, and in the female ovipositor (Fig. 2D), implying that these tissues may also have chemosensory roles in *C*. *hominivorax*.

### CRISPR/Cas9 gene editing efficiently generated *ChomOrco* null germline mutants

**Figure 3:**
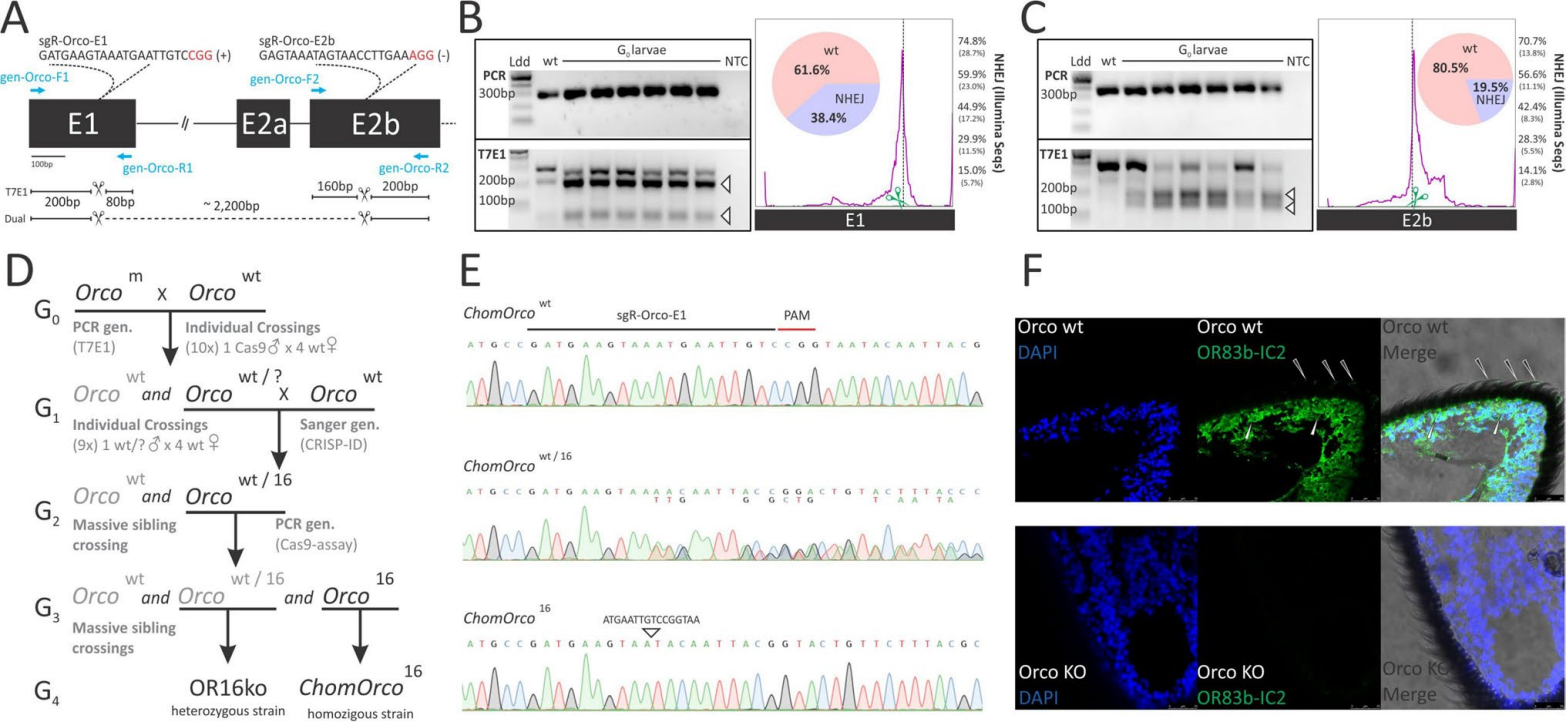
Knockout of *ChomOrco* by CRISPR/Cas9 induce complete loss of *Orco* protein in screwworm. (**A**) Schematic of the Cas9-targeted regions of *ChomOrco* and sgRNAs tested. Targeted sites are indicated by a scissor, PAM motif in red, and genotyping primers as blue arrows. Band migration patterns expected after T7E1 and dual-targeting assays are indicated below. (**B**,**C**) In vivo evaluation of sgR-Orco-E1 and sgR-Orco-E2b, respectively. A small number of surviving larvae (*n* = 6) were collected and genotyped by T7E1 (*leftmost*). PCR amplifications spanning targeted sites were pooled, Illumina sequenced, and genome modifications quantified using CRISPResso (*rightmost chart*). A diversity of allele variations is shown in Supplementary Fig. S3. Cropped images (delineated by black lines) of PCRs and T7E1 experiments are from samples run on the same gels (one for each experiment). Full-length gels are displayed in Supplementary Fig. S5. *wt* = wildtype sample; *NTC* = no template control; Ldd = 100 bp DNA Ladder (NEB). (**D**) Crossing scheme used to develop *Orco* mutant strains. Briefly, G_0_ flies were genotyped by T7E1, and mosaic males (*Orco*^m^) backcrossed to wt females (*Orco*^wt^). Heterozygous individuals (*Orco*^wt/?^) at G_1_ were genotyped by sequencing, and siblings harboring a − 16 bp mutation inbred at G_2_. Heterozygous (*Orco*^wt/16^) and homozygous (*Orco*^16^) flies at G_3_ were identified by in vitro Cas9-assay (Supplementary Fig. S8) and inbred to establish the heterozygous (OR16ko) and homozygous (*ChomOrco*^16^) strains. (**E**) Sequencing confirmation of the mutant genotypes in comparison with the wt allele. (**F**) Immunostaining of antennae sections from wt (*above*) and *ChomOrco*^16^ (KO, *below*) flies, showing cell nuclei (DAPI) and *Orco* protein (OR83b-IC3) localization within cell body (white arrows) and dendrites (black arrows) of screwworm’ OSNs. As in control slides (Supplementary Fig. S9), mutant flies display undetectable levels of *Orco* protein.

The evolutionary conservation of the *Orco* gene among dipterans might be translated into a conserved molecular function, suggesting that *ChomOrco* is required for normal OR-mediated olfaction in *C*. *hominivorax*. To test this assumption, we knocked out *ChomOrco* using CRISPR/Cas9 genome editing protocols we recently optimized for screwworm^23^. Initially, we tested in vivo two single guide RNAs (sgRNAs), targeting exons 1 (sgR-Orco-E1) and 2b (sgR-Orco-E2b) of the *ChomOrco* gene (Fig. 3A). Microinjections of pre-assembled ribonucleoproteins (RNPs), supplemented with a fluorescent protein-expressing plasmid, were performed in a small number of screwworm embryos (*n* ~ 100). Hatching fluorescent larvae (Supplementary Fig. S3) were examined for the presence of indels by T7 endonuclease 1 assays (T7E1), and induced genome modifications were quantified by Illumina sequencing (Supplementary Methods). Although both designed sgRNAs were found active, sgR-Orco-E1 outperformed sgR-Orco-E2b in induced mutagenesis (Fig. 3B,C). Indeed, microinjections with multiplexed sgRNAs poorly generated large deletion events between the targeted regions (Supplementary Fig. S4), most likely due to efficiency differences between designed sgRNAs.

Based on these initial results, new microinjections were carried out with sgR-Orco-E1 (*n* = 750). Out of the 329 hatching larvae (larvae surviving rate: 44%), a total of 154 showed expression of the co-injected fluorescent protein marker gene (putative microinjection success: 47%). Marked larvae were collected and reared until adulthood, and 28 healthy adults were obtained (adult surviving rate: 18%), including 12 males and 16 females. Only surviving G_0_ males were kept for backcrossing, as a high level of sterility has been previously observed for females developed from microinjections^23^. Non-lethal DNA extractions (Supplementary Methods and Supplementary Fig. S6) were used as templates for T7E1 genotyping, which revealed that all selected males harbored indels at the *ChomOrco* loci. Figure 3D summarizes the crossings and genotyping results obtained from this point. Ten males were randomly selected and individually backcrossed to virgin wild-type (wt) females to examine their founder capabilities. From each crossing, eight male-offspring (on average) were randomly sampled and genotyped as before. Out of the ten selected G_0_ putative founders, nine produced heterozygous G_1_ offspring (transmission efficiency: 90%) at percentages ranging from 14 to 89% (Supplementary Table S2), revealing germline Cas9-induced inheritable mutagenesis. Nine G_1_ heterozygous males (one per obtained line) were selected and individually backcrossed to wt females for a second time. Simultaneously, genotyping by sequencing was carried out, and resulting chromatograms were examined using the CRISP-ID application^42^ to access the allele variants harbored by each selected G_1_ male. In total, six mutated alleles were recovered, including three variants that disrupted the open reading frame of *ChomOrco*, and thus were expected to result in a non-functional *Orco* protein. Among them, siblings at G_2_ from a line harboring a − 16 bp deletion were selected and inbred. The − 16 bp mutation was chosen based on the likelihood of functional consequences, and due to the possibility of a simpler genotyping approach (Supplementary Fig. S7).

Finally, homozygous mutants were identified at G_3_ by in vitro Cas9-assay (Supplementary Fig. S8). Out of 96 genotyped G_3_ adults, 49 (51%) were heterozygous for the − 16 bp mutation (*ChomOrco*^16/wt^), 19 (20%) were wt (*ChomOrco*^wt^), and 28 (29%) were homozygous mutants (*ChomOrco*^16^), roughly as expected by the Mendelian inheritance ratio of genotypes (*X*^2^ = 0.4, *p* = 0.52, d.f = 1). Genotypes were confirmed by sequencing (Fig. 3E), and homozygous mutants inbred to establish a strain at G_4_. The *ChomOrco*^16^ mutants were evaluated for the loss of *Orco* protein expression by immunostaining. Polyclonal antibody IC3^27^ against the third intracellular loop of *Orco* labeled the OSNs cell body and dendrites inside the sensilla of wt flies’ antennae (Fig. 3F, *above*), while no labeling was detected in mutant flies (Fig. 3F, *below*). As in control slides (Supplementary Fig. S9), antennae sections of *ChomOrco*^16^ individuals lack detectable levels of *Orco* protein, confirming their knockout genotype.

### Disruption of *Orco* impairs foraging and host-seeking behaviors in *C. hominivorax*

Homozygous *ChomOrco*^16^ mutants do not exhibit any visible phenotype or locomotion disabilities, and they are fertile. However, we observed a clear reduction in overall fitness relative to the wt flies. The knockout strain was weak and difficult to rear in culture. Although these characteristics make screwworm *Orco* mutants unhandy for long-term rearing—for instance, the *ChomOrco*^16^ colony was maintained for eight inbreeding generations before collapsing—the heterozygous strain is as healthy as the wt, allowing to preserve the mutant allele in laboratory conditions. These observations indicate that *ChomOrco* might have a great impact in multiple traits of screwworm.

**Figure 4:**
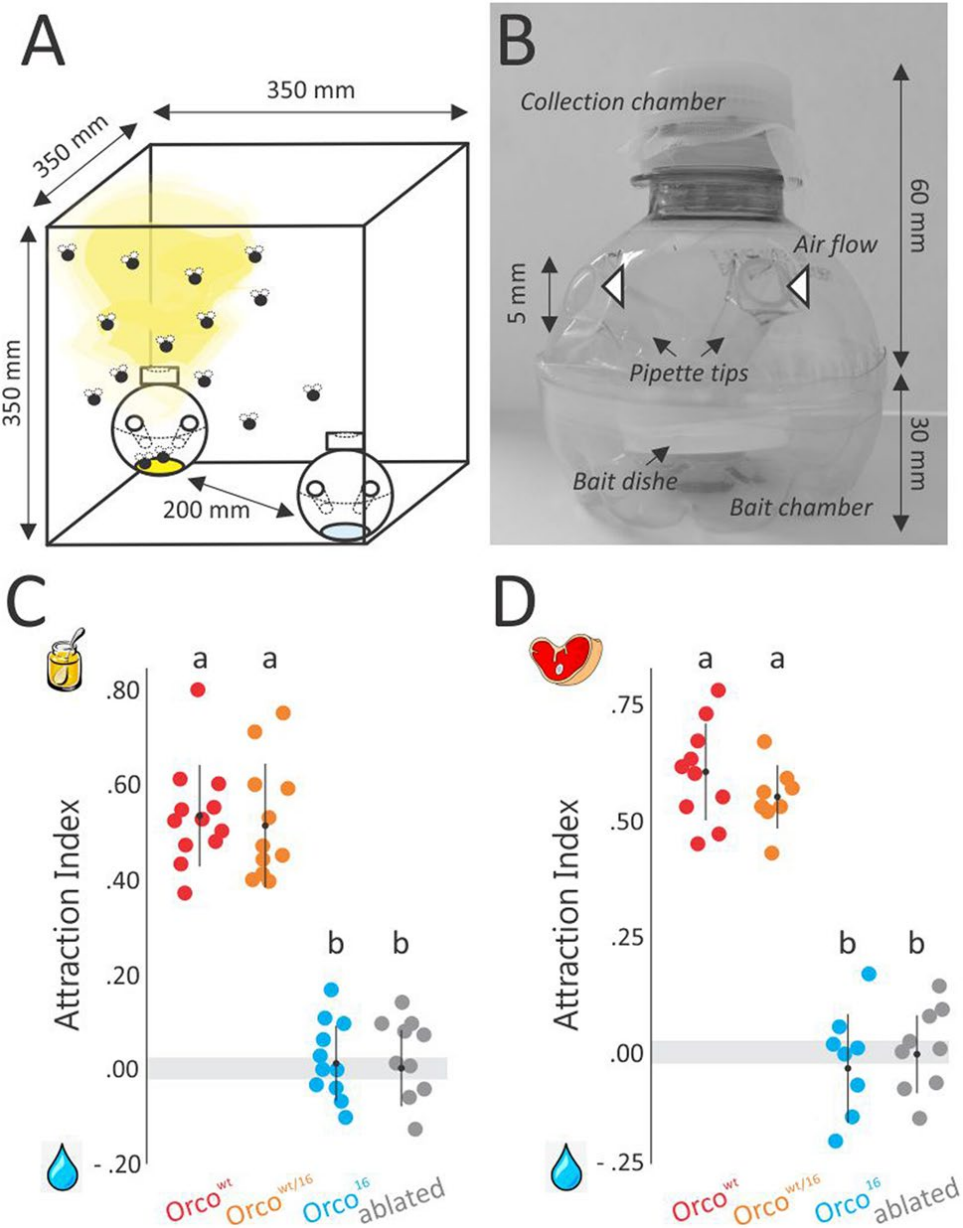
Disruption of *ChomOrco* impairs foraging and host-seeking behaviors in screwworm. (**A**) Representation of the two-choice trap assay arena. Odor (yellow mist) and control traps are offered to groups of flies at opposite sides of the cage. Groups of 15–25 flies (fasted adults of 3-to-4-days-old, sex ratio about 1:1) were tested in foraging trials, while groups of 10–20 flies (fed, matted females of 6-to-9-days-old) were tested in oviposition assays. Attraction experiments were made in replicates (*n* = 8 to 12; dots in **C**,**D**). (**B**) Details of the trap model used in two-choice assays (see also Supplementary Methods). (**C**) Honey odors resemble floral nectar, an important nutritional source for screwworm in nature. *Orco* mutants (Orco^16^; *n* = 11) lost attraction to honey, as ablated flies (*n* = 9), while wildtype (Orco^wt^; *n* = 12) and heterozygous (Orco^wt/16^; *n* = 12) flies remain strongly attracted. (**D**) Screwworm females are stimulated to lay eggs on spent larval media in rearing conditions. This bait releases a volatile blend similar to the one found in screwworm-infested wounds. *Orco* mutants (*n* = 8) have severely reduced attraction to the oviposition media when compared to wt (*n* = 10) and heterozygous (*n* = 8) flies (Supplementary Movie S1). Similar response was observed for ablated flies (*n* = 9). The results illustrate that OR-mediated olfaction is necessary for foraging and host-seeking behaviors in *C*. hominivorax. Bars indicate mean ± SD. Genotypes marked with different letters denote significant deviations of attraction index as given by the two-sample Wilcoxon rank-sum test with continuity correction (significant at *p*-value < 0.001).

We tested whether the mutation introduced in *ChomOrco* would translate into altered odor-guided behaviors in the screwworm by using two-choice trap assays (Fig. 4A,B), focusing on the adult foraging and host-seeking behaviors, as they are central for *C*. *hominivorax* ectoparasitic habit. Screwworm adults spend most of their time resting and feeding on flowering vegetation^43^. Nectar and pollen have a great impact on their survival, ovary maturation and reproductive success^44^. Hence, we tested the consequences of *Orco* disruption on the attractiveness of *C. hominivorax* male and female flies (3-to-4-day-old unmated and fasted flies) to honey (odor bait) or glycerol (control). As in other systems^32^, honey was used as an odor cue that resembles floral nectar. Control trials showed that wt flies don’t display preferences to cage sides, and they are not attracted to control baits (Supplementary Fig. S10). We found that as in ablated “*antennaless*” flies, screwworm *Orco* mutants exhibit no attraction to honey (attraction index (AI)_16_ = 0.01 ± 0.02, mean ± SEM), while heterozygous and wt flies showed a considerable preference for this nutritional source (Fig. 4C; AI_wt_ = 0.53 ± 0.03). In insects, members of the OR family are highly tuned to fruity ester and alcohol-derived smells^29^. Therefore, these results suggest that the disruption of *ChomOrco* most likely compromised the function of the *C*. *hominivorax* odorant receptors genes.

In nature, screwworm females are preferentially attracted by odors released from pre-existing screwworm-infested wounds^4,45,46^, as these sites are rich in bacterial-derived semiochemicals that act as animal host-finding cues in complex environments^18,19^. This blend of odors can be obtained from waste larval rearing media, which is routinely offered to *C*. *hominivorax* females to stimulate oviposition in laboratory colonies^47^. In order to evaluate the implications of an impaired OR-olfactory pathway for the recognition of its favored oviposition site, the ability of post-mated *C*. *hominivorax* female flies (6-to-9-days-old) to find oviposition media was evaluated in an additional set of two-choice trap assays. We observed that wt and heterozygous females display a strong attraction to the odors released from the oviposition device (Fig. 4D), entering the odor traps just a few minutes after the beginning of the trials and very often laying eggs inside before the end. In contrast, *ChomOrco*^16^ mutants showed drastically reduced attraction to the oviposition media (Wilcoxon test against wt: *W* = 80, *p* < 0.001), exhibiting a lack of decision-making and impaired flight orientation towards the stimuli source (Fig. 4D and Supplementary Movie S1). Comparable behaviors were observed for “*antennaless*” females. These outcomes demonstrate that *Orco* is required for normal host-seeking behavior in *C*. *hominivorax* and suggest that the perception of wound-derived odors by screwworm females relies, at least in part, on OR-mediated olfaction.

## Discussion

The emergence of the obligate ectoparasitic lifestyle in blowflies is a fascinating biological problem, which has been challenging scientists for over a century^7–13^. Surprisingly, few studies attempted to uncover the genetic basis of adaptive traits in this diverse group of flies. Since blowflies rely on odors to seek for oviposition sites, we hypothesized that changes in the olfactory system played a central role in the evolution of a free-living to a parasitic habit within the group. We adopted *C*. *hominivorax* as a model to expand our knowledge on the olfactory-driven behaviors in parasitic blowflies and focused our research on the characterization of the *Orco* gene, a required companion of all ORs. The *ChomOrco* is highly conserved within Diptera (Fig. 1A, Supplementary Fig. S1, and Supplementary Table S1), as a reflection of a strong purifying selection regime (Fig. 1B,C). Same signatures of sequence evolution were found in the distantly related species *D*. *suzukii* (Fig. 1C), an invasive phytophagous fruit fly. These observations indicate that other more divergent genes, rather than *Orco*, are responsible for the unique olfactory landscape of *C*. *hominivorax*. In fact, the functional conservation of *Orco* across ca. 290 Ma of insect evolution was illustrated by Jones et al.^48^. By using transgenic constructs, the authors demonstrated the feasibility of using *Orco* orthologs from close and distantly related species to rescue functional defects in *Orco* mutants of *Drosophila*, clearly demonstrating the critical importance of this gene for insect olfaction.

Nevertheless, the evaluation of *ChomOrco* expression allows us to speculate on the contribution of olfaction to many aspects of screwworm biology. Quantitative analysis indicated that the olfactory system changes drastically throughout development in *C*. *hominivorax* (Fig. 2A). *Orco* transcription appears at the last hours of embryogenesis, persisting during the first larvae stage. Screwworm larvae survival depends on proper host selection made by the mothers, which prefer to lay eggs on dry borders of animal wounds and bodily orifices^46^. This decision prevents the embryos from drowning in body fluids while ensuring hatching larvae immediate access to a nutrient-rich environment. Newborn larvae might use olfactory cues to guide their way from the oviposition site into the substrate to feed. Once the feeding source is found, this olfactory-based orientation might be gradually replaced by contact chemoreception, such as gustatory, explaining the decrease in *ChomOrco* expression during subsequent larval stages (Fig. 2A). A similar pattern is observed in other blowflies, such as *Lucilia sericata*^49^ and *C*. *megacephala* (Fig. 2C), indicating that the modulation of *Orco* expression is also evolutionary conserved. Interestingly, food ingestion by *D*. *melanogaster* is enhanced in the presence of microorganism-derived odors^50^. Thus, a reduction in olfactory input might be related to the lower rates of larval survival observed for the *Orco* mutant strain developed in this study.

The relatively low expression of *Orco* during larval stages also reflects their morphologically simple olfactory system^51^. In fact, *ChomOrco* expression appears to be in synchrony with the morphogenesis of the adult peripheral olfactory system (Fig. 2A,B). The correlation between structure and function, in this case, is illustrated by the high expression of *ChomOrco* in the antennae and maxillary palps of both sexes (Fig. 2D,E). Olfaction is indispensable for *C*. *hominivorax* adulthood, in particular to locate susceptible animals for oviposition^43,52, 53^. Animal host-finding and selection by screwworms can be separated in three scalable steps, from initial activation to orientation, and landing, culminating in the final decision of egg-laying. As gravid screwworms approach putative animal hosts, visual cues encourage the selection of landing sites, and other systems such as tactile, gustatory, and thermal contribute to generating enough stimuli for the egg-laying decision^53^. As in other Calyptratae flies^39,49^, *Orco* transcription is detected in screwworm legs and abdomen of both sexes, and in the female ovipositor (Fig. 2D), suggesting that *ChomOrco* has a broader chemosensory role in *C*. *hominivorax*. Additionally, these appendages are likely to harbor unknown olfactory structures in the screwworm, as widely described in other species^39,54–56^, which in females would enhance the acquisition of short-range and contact stimuli required for oviposition^45,52^. Indeed, we noticed that *Orco* mutants invest considerably more time examining oviposition devices in comparison to wt flies, often laying smaller batches of eggs, presumably due to their chemoreception disabilities.

Odors emitted from wounded animals are perceived by gravid screwworm females through receptors located in the antennae, which are responsible for eliciting flight orientation towards putative animal hosts^18,19,52,53^. This was first demonstrated by Devaney et al.^52^, which found that the removal of screwworm females’ antennae is enough to disrupt their host-seeking behavior. This finding was subsequently confirmed by olfactometer assays^57^, and corroborated by the characterization of screwworm’s antennae ultrastructures^41^. Here, we present a further step in dissecting the behavioral responses mediated through olfactory receptor families in screwworm, by developing a germline *Orco* null strain of *C*. *hominivorax* using CRISPR/Cas9 genome editing (Fig. 3). Without *Orco*, the correct localization of all ORs is compromised, preventing the OR-signaling while maintaining other chemosensory pathways unharmed^26,27,29,30^. This knockout system allowed us to evaluate the contribution of OR and IR olfactory receptor families on *C*. *hominivorax* foraging and host-seeking behaviors using two-choice trap assays (Fig. 4A,B). While wt and heterozygous flies are highly attracted to both honey and oviposition devices, the disruption of *Orco* in screwworm resulted in a lack of decision-making and impaired flight-orientation, suggesting they were unable to perceive either smell (Fig. 4C,D, and Supplementary Movie S1). Furthermore, experiments with ablated individuals revealed that other chemosensory appendages do not compensate for the absence of the antennae to elicit flight-orientation towards these odors. The consequences of *Orco* disruption on specific traits have been described in a number of non-classical model insects, including social behavior in ants^34^, conspecific recognition in locust^58^, foraging and mating in moths^33,35,59^, human host-seeking in mosquito^32^, and oviposition in the spotted wing *Drosophila*^37^. Together, these studies unlock the major role of the OR gene family on insect evolution. Likewise, our results demonstrate that OR-mediated olfaction is required, at least in part, for normal screwworm host-seeking behavior, by inducing flight-orientation towards animal host-derived odors. They further illustrate one of many potential roles that *Orco* and ORs play in the ectoparasitism habit of the screwworm fly. Consequently, changes in the OR-olfactory pathway are expected to have contributed to shaping the ecological niche preference in the *C*. *hominivorax* lineage during blowfly evolution.

In the past decade, there has been great progress in the development of genomic tools supporting investigations on screwworm biology, including transcriptome sequencing^60–62^, molecular tools enabling gene silencing, editing and engineering^23,63,64^, and more recently the first reference genome assembly^24^. Together with this increasing availability of genomic resources, the results presented here set the stage for a broader investigation focusing on the genetics underlying specific traits in screwworm. It will be possible, for example, to search for signatures of diversifying selection on chemosensory gene families and evaluate their expression profiles at contrasting *C*. *hominivorax* physiological states. Supporting this idea, a recent study^65^ found that screwworm females and males exhibit very distinct antennal expression profiles, including many genes encoding for ORs. The study shows that at least one OR displaying a female-biased expression might have a role in the screwworm oviposition behavior. The *ChomOr19* gene, as named by the authors, is an ortholog to the *DmelOr7a*; an OR related to aggregation and egg-laying decision in *D*. *melanogaster*^66^. Functional analysis of *ChomOr19* and other genes of interest will benefit from our CRISPR/Cas9 methods developed for screwworm, allowing us to investigate their contribution to the different life strategies adopted by blowflies. From an applied perspective, these efforts may assist in the identification of novel targets for alternative genetic strategies aiming to interfere with specific screwworm behaviors, in addition to the discovery of new environmentally safe semiochemicals to be used in integrated pest management programs^67^. We have just begun a deep dive into the genetic basis of niche specialization in *C*. *hominivorax*, a species that is on its way to become a compelling model organism to study the link between chemoperception evolution and the emergence of novel ecological adaptations in the Calliphoridae family.

## Methods

### Flies

The *C. hominivorax* wt strain J06 was used in this study. The strain is routinely reared in the Agricultural Research Service (ARS) laboratory, located inside the Commission for the Eradication and Prevention of Screwworms (COPEG) biosecurity plant in Panama, under conditions previously described^63^. Samples of *Cochliomyia macellaria* and *C. megacephala* were obtained from the frozen tissue collection of the Department of Genetics, Evolution, Microbiology and Immunology at the University of Campinas (UNICAMP), Brazil.

### RNA and cDNA

Specimens were collected from colonies, while specific tissues were dissected from 3-days-old adult flies fasted for 12 h. Samples were rinsed with 0.1% DEPC-treated water and immediately homogenized in TRIzol reagent (Invitrogen). Total RNA was isolated according to manufacturer’s instructions. All extractions were DNase-treated in a 20 µl reaction containing 4 U of TURBO DNase (Invitrogen), 20 U of RiboLock RNase Inhibitor (Thermo Scientific), 1X Reaction Buffer and 10 µg of total RNA. Treatments were performed at 37 °C for 30 min and stopped by the addition of 15 mM of EDTA (pH 8.0) followed by an incubation at 75 °C for 10 min. To ensure complete digestion of genomic DNA, a total of 1 µg of each RNA preparation was visualized in a 1% denaturing agarose gel (1X MOPS, 2% formaldehyde) post-stained with Ethidium Bromide (EtBr; 0.5 µg/ml). Samples were previously mixed with 2X volume of Gel Loading Buffer II (Invitrogen), denatured at 65 °C for 20 min, and let cool down in ice for 5 min before gel applications. First-strand cDNAs were synthesized from 2 µg of DNase-treated RNA using the SuperScript II (Invitrogen) protocol with the Oligo(dT)_12–18_ primer (Invitrogen). Reverse transcriptions were performed at 42 °C for 1 h, terminated at 70 °C for 15 min, and stored at − 20 °C.

### RACE and genomic region

Specification for all primers used in this study can be found in Supplementary Table S4. First strand cDNA was used as template for the amplification of a 660 bp region of *Orco* transcripts from *C*. *hominivorax* (*ChomOrco*) and *C*. *macellaria* (*CmacOrco*; used as a control in this study) in a 50 µl PCR reaction containing 0.2 µM of each Orco-F1 and Orco-R1 primers, 80 µM of dNTPs, 10% Bovine Serum Albumin (BSA at 5 mg/ml), 1X Taq buffer supplied with 1.5 mM of MgCl_2_ and 1.25 U of recombinant *Taq* DNA polymerase (Invitrogen). Amplifications were carried out in the following conditions: 95 °C for 3 min, 35 cycles of [95 °C for 30 s, 60 °C for 45 s and 72 °C for 60 s], and a final extension at 72 °C for 5 min. PCR products were purified using the QIAquick PCR Purification Kit (Qiagen), directly TA cloned into a pGEM-T Easy Vector (Promega) and sequenced in a 3730xl DNA Analyzer (Applied Biosystems) using the universal primers M13-Forward and M13-Reverse. Obtained sequences were used to design specific inner primers. Classic rapid amplification of cDNA ends (Classic-RACE) was performed as described by Scotto–Lavino et al.^68,69^ with minor modifications described in Supplementary Methods. The resulting *ChomOrco* transcript was mapped against the *C*. *hominivorax* genome (GenBank: GCA_004302925.1) using tBLASTn, and exon–intron boundaries predicted using exonerate^70^. The resultant genomic map was used to design specific primers for long-range PCRs (long-PCR), which were carried out to fill sequence gaps and validate complex regions through Long-PCR Product Sequencing (LoPPs) method^71^ (Supplementary Material). Previously assembled *ChomOrco* transcript was used to anchor LoPPs reads during the final assembling with CAP3^72^.

### Evolutionary analysis

Selected Diptera *Orco* sequences (Supplementary Table S3) were codon-aligned using the Muscle algorithm implemented in MEGA X^73^. Neighbor-Joining (NJ) method was used to estimate uncorrected distances (*p*-distance; Supplementary Table S1). Maximum-likelihood (ML) reconstructions were made in RAxML v.8^74^ using the PROTGAMMA model and JTT substitution matrix for the amino acid alignment. Node supports were assessed by 500 non-parametric bootstrap replicates. The clade *Aedes aegypti* + *Anopheles gambiae* was defined as the outgroup. Adaptive evolution was tested using CodeML implemented in PAML4^75^. Normalized non-synonymous (d_N_) to synonymous (d_S_) substitution rates (ω) were estimated using branch-site models to detect events of episodic selection on amino acid sites at specific lineages (foreground branches). Likelihood ratio tests (LRTs) were performed between the alternative model bsA (positive selection) and the null model bsA1 (neutral). Significance of LRT results were determined by chi-squared (*χ*^2^) testes, and Bayes Empirical Bayes analysis (BEB) was used to infer amino acid sites under selection regime (cutoff ≥ 0.95). Consensus locations of transmembrane domains (TM) within *ChomOrco* were predicted by TOPCONS^76^, and significantly characterized sites were mapped onto the predicted protein topology as modeled in Protter^77^.

### Expression

Evaluation of *ChomOrco* expression was conducted by Quantitative Real-Time PCR (qPCR). First-strand fivefold diluted cDNAs (equivalent to 50 ng of DNAse-treated RNA template, as determined by 1:5 serial dilution standard curve analysis) were used as templates in 12.5 µl amplification reactions containing 6.25 µl of SYBR Green PCR Master Mix (Applied Biosystems), and 0.4 µM of each Orco-F2 and Orco-R2 primer (Supplementary Table S4). Runs were performed in a StepOnePlus Real-Time PCR System (Applied Biosystems) according to the following protocol: hold at 50 °C for 2 min and 95 °C for 10 min, followed by 40 cycles of [95 °C for 15 s and 62 °C for 60 s]. Amplification efficiency was evaluated by standard curve method, and melting curves were assessed to ensure unique product amplification (Supplementary Fig. S11). Data were analyzed with automatic threshold and baseline settings. The expression levels of *ChomOrco* during the screwworm development were normalized to *Gapdh*^78^ using the 2^-ΔCt^ method, and presented as fold-change relative to the third instar larvae using the 2^-ΔΔCt^ method^79^. Cycle thresholds above 35 were considered undetected. Independent biological (*n* = 3) and technical (*n* = 3) replicates were carried out for each experiment, in addition to no template controls (NTC, cDNA omitted from the reaction and volume adjusted with nuclease-free water). Tissue-specific semi-quantitative amplifications were made with primers Orco-F3 and Orco-R1 (Supplementary Table S4) in replicates (*n* = 3) through Reverse Transcription PCR (RT-PCR). First strand cDNAs were used as templates in 25 µl PCRs as previously described, and amplifications were resolved in 1X TAE (40 mM Tris–acetate, 1 mM EDTA) 2% agarose gels post-stained with GelRed (GLPBIO). Gel images were acquired in a L-PIX EX transilluminator (Loccus Biotecnologia) using pre-defined setups in the software L-Pix Image v.2.7. Cropping, contrast and light corrections were made in the same software.

### CRISPR/Cas9

CRISPR experiments were performed as previously described^23^. Single guide RNAs (sgRNAs) were designed by examining *ChomOrco* exons for the presence of protospacer-adjacent motifs (PAMs, sequence NGG-3’, where “N” is any base) using the standalone version of CRISPOR tool^80^ in the context of *C*. *hominivorax* genome (GenBank: GCA_004302925.1). The sgRNAs were synthesized as described by Bassett and Liu^81^, with minor modifications^23^, while purified recombinant Cas9 protein was obtained commercially (PNA Bio). Ribonucleoprotein complexes (RNPs) were pre-assembled by incubating Cas9 protein (500 ng/µl) with specific sgRNA (200 ng/µl) in a Sodium Phosphate Buffer (supplied with 300 mM of KCl) at 37 °C for 10 min. For multiplexed experiments, RNPs were pre-assembled separately and 1:1 mixed prior to the microinjections. The plasmid pB[Lchsp83-ZsGreen]^82^ was added to the final injection cocktail (300 ng/µl), which was maintained on ice during the experiments. Microinjections were performed at the posterior end of pre-blastoderm screwworm embryos within the first 45 min of embryogenesis.

### *Orco* mutant strain

After microinjections, all G_0_ larvae transiently expressing the ZsGreen marker were selected and raised to adulthood. Non-lethal DNA extractions (Supplementary Fig. S6 and Supplementary Methods) were performed for G_0_ adult flies and used as templates for PCR amplifications spanning the targeted site at *ChomOrco* exon 1. Amplifications were submitted to T7E1 cleavage assays, as previously described^23^, and ten mosaic males were individually backcrossed to wt females (1 Cas9 G_0_ ♂ × 4 wt ♀). On average, eight G_1_ adult males from each crossing were randomly selected and genotyped as before. Nine heterozygous G_1_ males (one per obtained mutant line) were selected and individually backcrossed to wt females for a second time, while simultaneously genotyped by Sanger sequencing and CRISP-ID analysis^42^. Adult G_2_ male and female flies harboring a − 16 bp deletion were identified by T7E1 and let to interbreed freely in cages. Homozygous mutants at G_3_ were identified by in vitro Cas9-assay, using the Guide-it Genotype Confirmation Kit (Takara), and inbred to establish the *Orco* mutant strain at G_4_. Further genotyping, when required, was conducted using our custom High Resolution Mobility analysis (HRMob, described in Supplementary Fig. S7).

### Immunostaining

Antennae were dissected from females and directly frozen in O.C.T. Compound (Sakura Tissue-Tek) at − 20 °C. Cryosections were made in a Leica CM1850 at a thickness of 18 µm, and thaw-mounted on gelatin-coated microscope slides. Slides were air-dried at room temperature for 10 min, submerged in phosphate-buffered saline (PBS) supplemented with 0.05% Azide for 5 min, and blocked in PBS with 0.2% Triton X and 3% Bovine Serum Albumin (PBSTB) for 1 h. Sections were incubated with anti-Or83b (peptide HWYDGSEEAKT, described in^27^) rabbit polyclonal antibody (1:100 in PBSTB) overnight at 4 °C in a humid chamber. Slides were washed for a total of 15 min in PBST (5 min per wash) and incubated at room temperature for 1 h with donkey anti-rabbit Alexa 488 secondary antibody (Life Technologies, diluted 1:500 in PBSTB). Slides were washed as before, with the addition of 4′,6-diamidino-2-phenylindole (DAPI) during the last wash, mounted using the Vectashield medium (Vector Labs), and sealed with nail polish. Stained antennae sections were imaged on a Leica TCS SP5 II confocal microscopy at the Life Sciences Core Facility (LaCTAD), located at UNICAMP. The wt and mutant samples were mounted in the same slides and imaged under the same settings. No signal was detected in the absence of primary or secondary antibodies (Supplementary Fig. S9).

### Behavior

For the foraging assays, unmated adult flies of 3-to-4-day-old were fasted for 12 h with access to water, transferred without anesthesia to the test arena (BioQuip Inc., model 1450B), and left to acclimate for 15 min before the trials. Groups of 15 to 25 flies (males and females, ratio about 1:1) were tested in each trial. Odor bait consisted of 400 mg of natural honey (handcrafted at Holambra-SP, Brazil) applied on a 25 mm diameter Whatman filter paper (GE Healthcare), while the control bait contained the same weight of glycerol (Sigma-Aldrich). Two-choice assays were performed under controlled conditions (25 ± 2 °C, 65 ± 5% RH, 12:12 L:D) during the morning (from 7 to 11 am), as favored by screwworm adults^43^. Testing cages were positioned below fluorescent lights and side-enfolded with white paper sheets to provide a homogeneous light distribution from above. Control trials ensured that flies did not prefer one side of the cage upon the other (Supplementary Fig. S10). Ablated “*antennaless*” flies (i.e., adults that had at least the 3rd antennae segment physically removed the day before trials) were also tested in order to ensure that choices were guided by olfaction. For the oviposition assays, groups of 10 to 20 fed and post-mated adult females of 6-to-9-day-old were tested in each trial. Odor bait contained 10 ml of 25% warmed waste larval rearing media^47^, while the same volume of warmed distilled water was used as control. Oviposition assays were performed in complete darkness, as it appears to increase oviposition attraction^45^ while also avoiding visual cues. Traps (Fig. 4B, see Supplementary Methods for craft details) were alternatively placed in opposite sides of cages, and their positions altered for every trial. Captures were scored after 4 h (foraging) or 90 min (oviposition). Attraction index was calculated as: AI = (n_odor_ − n_control_)/n_total_, where n_odor_ is the number of flies captured in the odorant trap, n_control_ the number of flies captured in the control trap, and n_total_ the total of flies tested. The AI ranges from − 1 (complete avoidance) to + 1 (complete attraction). Values of zero characterizes a neutral or non-detection of odors. Significant deviations of AI were tested with the two-sample Wilcoxon rank-sum test with continuity correction using the *wilcox.test* function implemented in the R/stats package.

## Supplementary Information

Supplementary Video 1.

Supplementary Information 1.

## Data Availability

The *ChomOrco* sequences were submitted to GenBank and can be retrieved under the accession numbers MT226797 and MT226799, for transcript and genomic sequences, respectively. The *CmacOrco* transcript can be retrieved under the GenBank accession number MT226798. A stable screwworm heterozygous population for *ChomOrco* (named Orco16Ko) is being maintained at the ARS laboratory at COPEG in Panama.
