# Supplementary material for: Disruption of the odorant coreceptor Orco impairs foraging and host finding behaviors in the New World screwworm fly
Source: Sci Rep. 2021 May 31;11:11379. doi: 10.1038/s41598-021-90649-x (PMC8167109; doi:10.1038/s41598-021-90649-x)
Supplement: Supplementary file 2 — Supplementary Information 1. [file 41598_2021_90649_MOESM2_ESM.pdf]

Supplementary Material for

Disruption of the odorant coreceptor *Orco* impairs foraging and host finding behaviors in the New World screwworm fly

Daniel F. Paulo, Ana C. M. Junqueira, Alex P. Arp, André S. Vieira, Jorge Ceballos, Steven R. Skoda, Adalberto A. Pérez-de-León, Agustin Sagel, William O. McMillan, Maxwell J. Scott, Carolina Concha* and Ana M. L. Azeredo-Espin

* Correspondence: conchamc@si.edu (C.C)

Content:

Supplementary Figs S1 - S11
Supplementary Tables S1 - S4
Supplementary Methods
Supplementary References

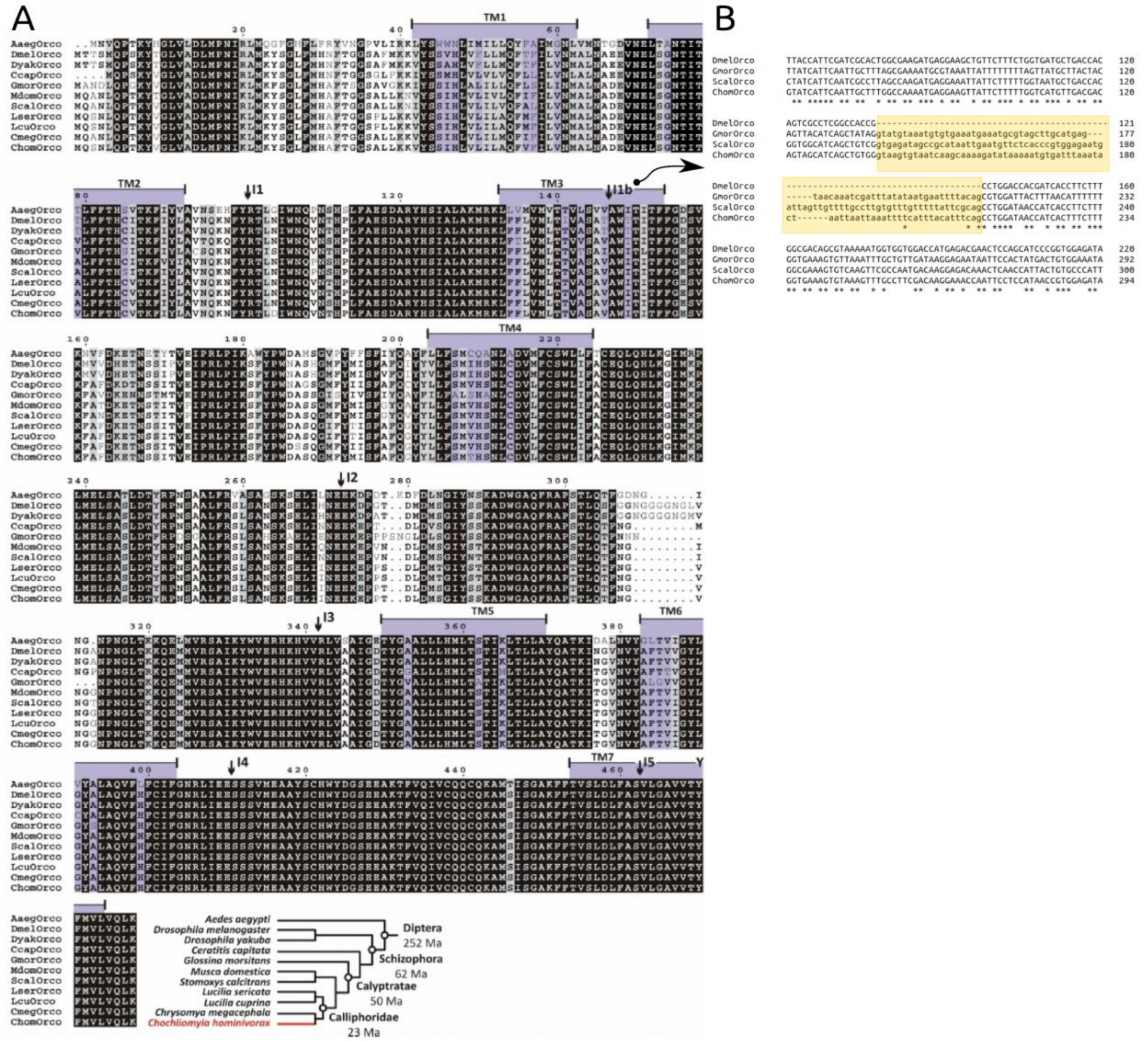

Supplementary Fig. S1 (**A**) Multiple sequence alignment of *Orco* orthologues in Diptera. Codon-based alignment was constructed using the Muscle algorithm implemented in MEGA7 [1]. Black arrows indicate the locations of the six introns identified in the *ChomOrco* genomic region. Purple boxes indicate the positions of the seven transmembrane domains (TM1 to TM7), as predicted by TOPCONS [2]. Alignment is shown in the context of the evolutionary relationships among Diptera species, according to Junqueira et al. [3]. Species abbreviations and accession numbers are given in Supplementary Table S3. (**B**) Partial alignment of *Orco* exon 2 genomic regions from *D. melanogaster* (FlyBase: FBgn0037324), *G. morsitans* (VectorBase: GMOY005610), *S. calcitrans* (GenBank: JX996042.1), and *C. hominivorax* (this study), showing the ancient origin of intron I1b (yellow box) in Calyptratae flies.

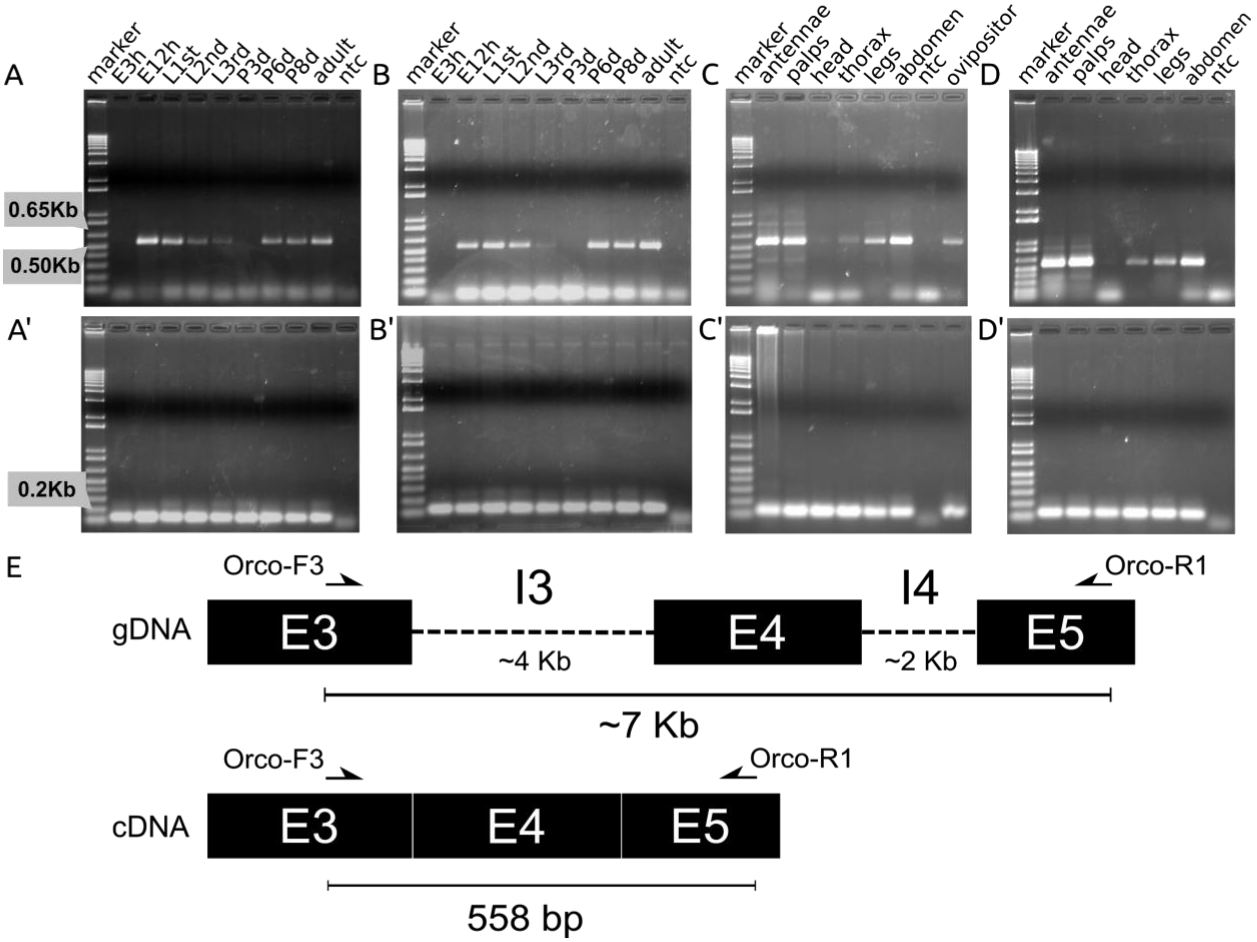

Supplementary Fig. S2 Original full-length images of the cropped gels shown in Fig. 2C and 2D. Amplifications of *Orco* in *C. hominivorax* (**A**) and *C. megacephala* (**B**) during their development. *GAPDH* was amplified from the same cDNA samples as an internal control (**A'** and **B'**). Amplifications of *ChomOrco* in different tissues of adult screwworm female (**C**) and male (**D**). *GAPDH* was amplified from the same cDNA samples as an internal control (**C'** and **D'**). Gel labels according to Fig. 2. Marker = 1 Kb Plus DNA Ladder (Invitrogen). Primers Orco-F3 and Orco-R1 were used for Orco amplifications, while Gapdh-F1 and Gapdh-R1 were used for *GAPDH* amplifications (see Supplementary Table S4). (**E**) Amplification scheme of the exon-spanning strategy used for RT-PCRs, showing expected sizes for genomic DNA (gDNA) and complementary DNA (cDNA) amplifications of *ChomOrco* targeted sequences.

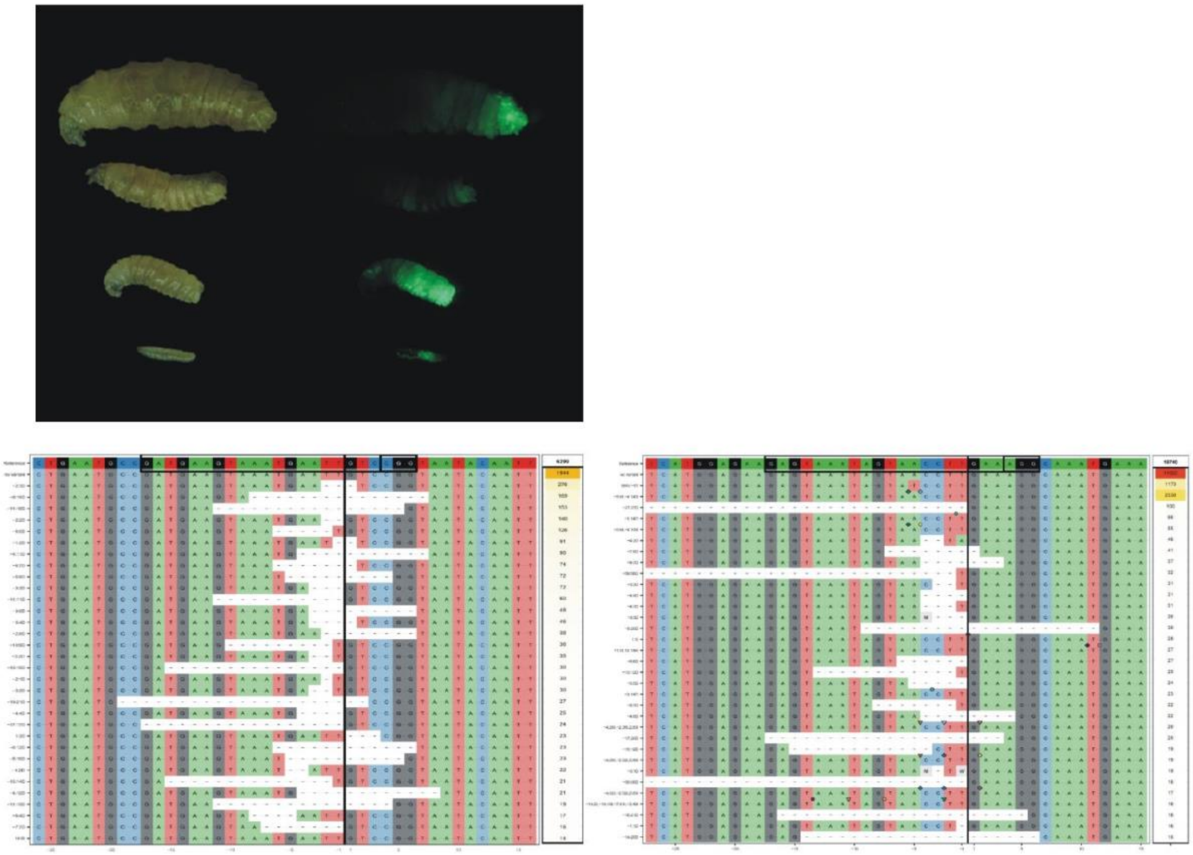

Supplementary Fig. S3 (*above*) Screwworm larvae transiently express the ZsGreen marker during all its development stages, as observed in bright (*left*) and fluorescent (*right*) light. (*below*) Allele variants sampled by Illumina sequencing of flies microinjected with sgR-Orco-E1 (*leftmost*) and sgR-Orco-E2b (*rightmost*), referent to results shown in Figure 3. Allele examinations were conducted using the CrisprVariants pipeline [4]. Only top *n* = 35 most frequent alleles are shown. Amplifications of *ChomOrco* exon 1 and exon 2b (Fig. 3A) were conducted using the primers genOrco-F1 along with genOrco-R1, and genOrco-F2 along with genOrco-R2, respectively (Supplementary Table S4).

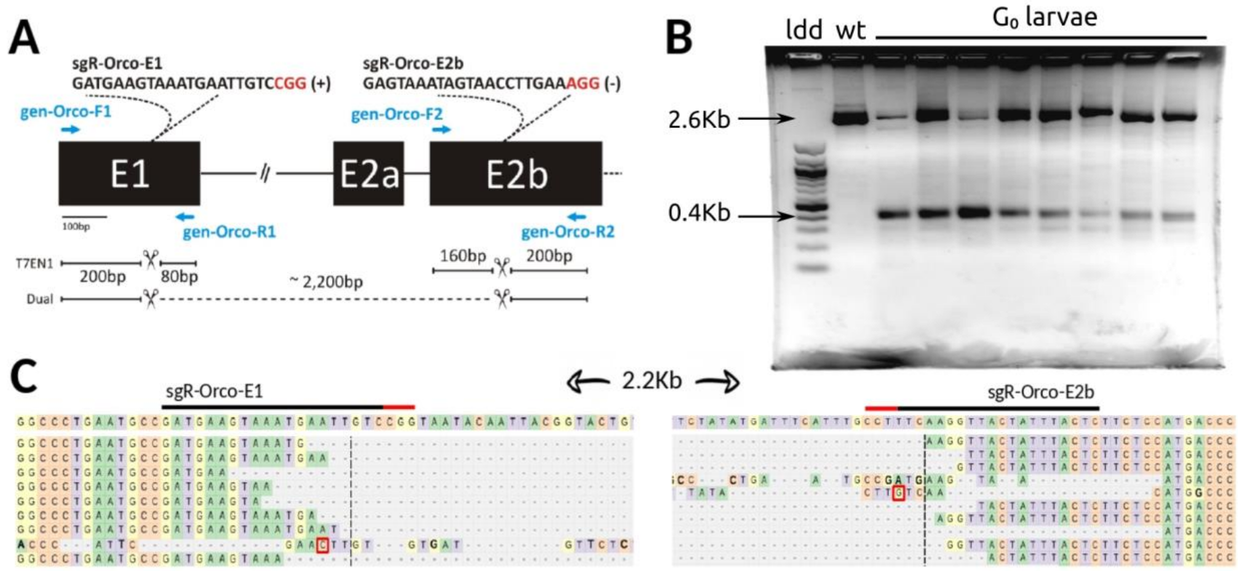

Supplementary Fig. S4 CRISPR/Cas9 dual-targeting approach. In addition to the PCR-based genotyping methods used, we evaluated the possibility of inducing large deletion events between Cas9 targeted regions by injecting RNPs multiplexed with both sgRNAs. (**A**) The goal was to introduce a ~2.2 Kb deletion at the *ChomOrco* loci between sgR-Orco-E1 and sgR-Orco-E2b targeted sites. (**B**) Large deletions would be easily distinguished from the wildtype (wt) allele by routine PCR, and electrophoresis. A degree of deletion events was observed from G_0 individuals developing from dual-targeting microinjections, but with low efficiency. Complete ˜2.2 kb deletions were rarely seen, and none large deletions were observed after crossing dual-targeting survivors with wt flies. These results may reflect the unbalanced efficiency of the designed sgRNAs (referent to results in Fig. 3). Abbreviations used: wt = wildtype sample; Ldd = 100 bp DNA Ladder (NEB). Primers genOrco-F1 and genOrco-R2 were used (see Supplementary Table S4). (**C**) Sequencing confirmation of target-specific deletions between exons E1 and E2b of *ChomOrco*.

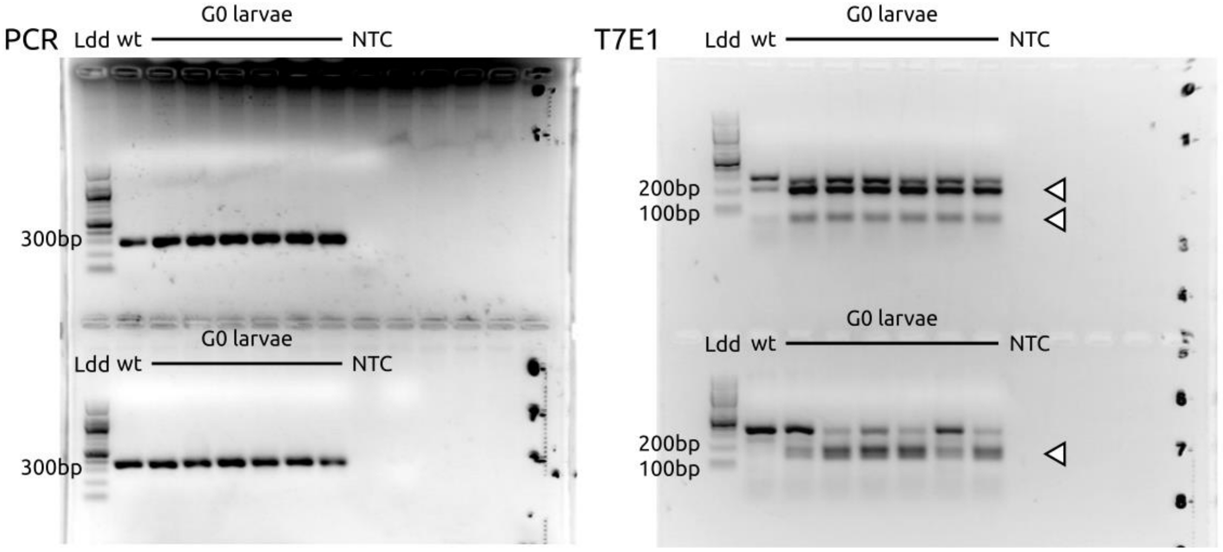

Supplementary Fig. S5 Original full-length images of the cropped gels shown in Fig. 3B and 3C. Figure labels according to Fig. 3. Abbreviations used: wt = wildtype sample; NTC = no template control; Ldd = 100 bp DNA Ladder (NEB). Amplifications of *ChomOrco* exon 1 and exon 2b (Fig. 3A) were conducted using the primers genOrco-F1 along with genOrco-R1, and genOrco-F2 along with genOrco-R2, respectively (Supplementary Table S4).

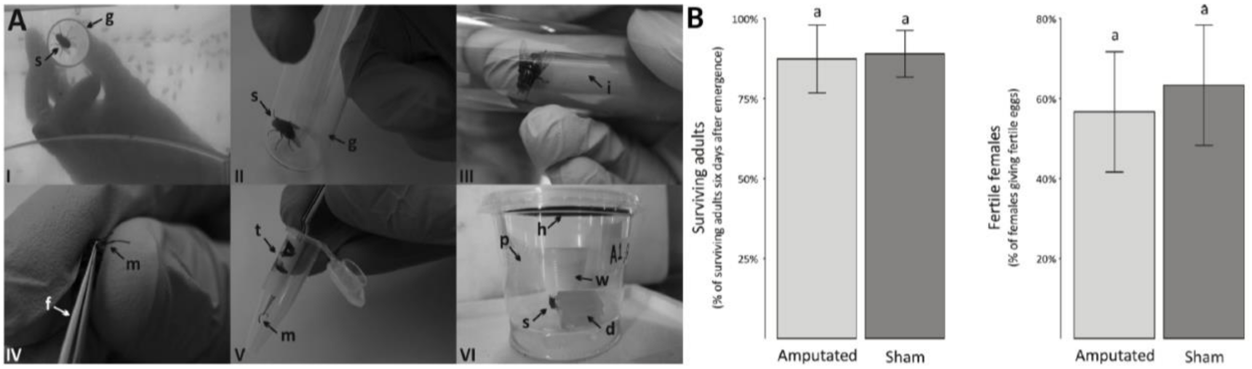

Supplementary Fig. S6 Non-lethal tissue sampling for DNA isolation. (**A**) Upon emergence, adult flies are individually captured in a 30 mL glass vial (*panels* I and II). The captured flies are gently held and turned upside down to expose their legs (*panels* III and IV). A single midleg is then sectioned at the proximal coxa using a fine forceps (*panel* IV). Legs are transferred to a 1.5 mL centrifuge tube and maintained at -80° until DNA extractions (*panel* V), while adults are transferred to an individual plastic cage provided with food and water (*panel* VI). (**B**) To evaluate the amputation effects on survival and fertility, dissected adult flies were transferred from the individual cages to mating assay cages (three days after leg amputation). Test cages (n = 6) were founded with ten amputated individuals each (males and females, ratio 1:1), while sham individuals (flies that passed through the same sampling procedure but dissection) were used in control cages (n = 6). Survival rates were evaluated six days after adult emergence, and females were individually stimulated to oviposit inside 30 mL glass vials. Viable offspring production was checked on the next day. Bars marked with the same letters are not significantly different as given by two-tailed Student's t-test. Results showed that the non-lethal tissue sampling has little or no hazardous effects on screwworm survival and fertility. Abbreviations used: s (screwworm); g (glass vial); i (index finger); f (forceps); m (midleg); t (tube); d (diet); w (water); p (gauze perch); h (holes). Photo credit to Stephanie Mladinich.

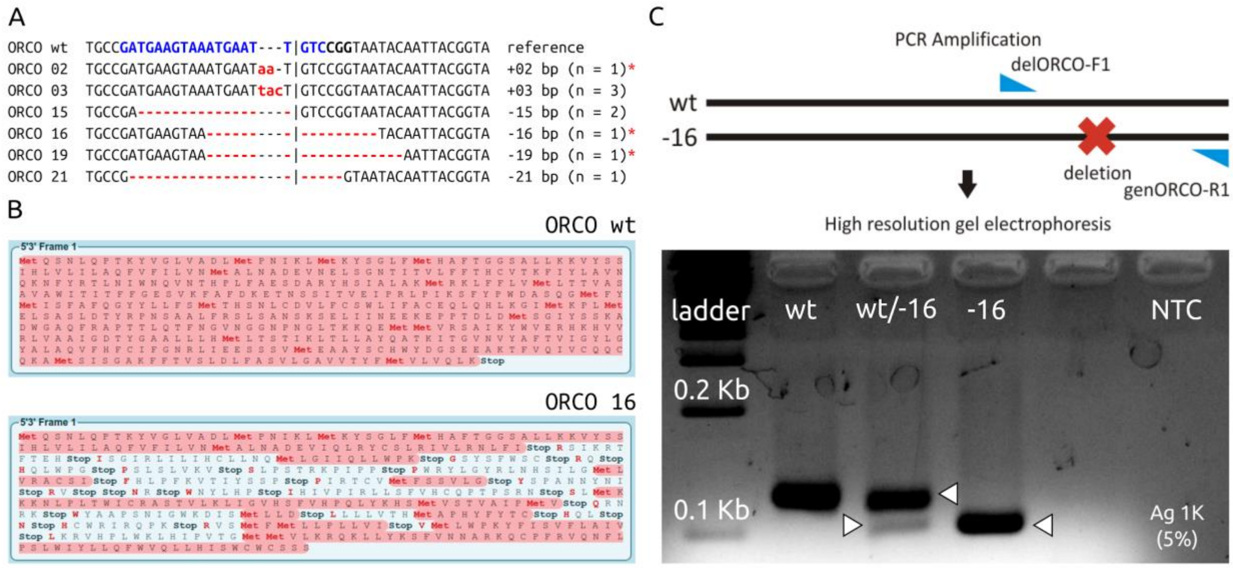

Supplementary Fig. S7 Selection of mutant allele at G_1. (**A**) Cas9-induced mutated alleles in *ChomOrco* obtained at G_1. The sgR-Orco-E1 sequence is highlighted in blue letters (in the context of wt reference sequence), the PAM motif in bold black letters, and the putative Cas9 cleavage site in vertical dashes. Sequence modifications are highlighted in red letters (insertions) and in red dashes (deletions). The number of individuals carrying the specified allele is given in parenthesis. Mutated alleles producing premature stop codons within the *ChomOrco* coding sequence are designated with asterisks (*). (**B**) Consequence of the -16 bp deletion at *ChomOrco* loci. Among the developed lines, the one harboring a -16 bp deletion was selected and inbred to establish a homozygous mutant strain. This mutation was chosen based on the likelihood of functional consequences on *ChomOrco*, as it introduces several premature stop codons on the *Orco* transcript preventing the translation of a functional protein. The putative founder carrying a similar mutation (-19 bp) did not produce viable eggs. Coding sequences were translated using the ExPASy translate tool [5]. (**C**) Genotyping by High Resolution Mobility analysis (HRMob). For the HRMob, a short region (≤ 200 bp) spanning the mutated region of *Orco* exon 1 is amplified via PCR using the primers delOrco-F1 and genOrco-R1 (Supplementary Table S4). Amplifications are resolved in a high-resolution 5 - 6% Agarose-1000^{TM} (Invitrogen), prepared in 1x Tris-Borate-EDTA (TBE) buffer and stained with 3 µl of ethidium bromide (10 mg/mL). Samples are mixed with 3 µl of Gel Loading Dye (NEB) and loaded into the gel. Electrophoresis is performed for at least 2 h at 5 volts/cm in a cold 1x TBE buffer. The gel image displays an example of the expected results from the HRMob analysis using control samples. Only the informative area of the gel is shown; from the top wells of the gel to the last band in the molecular marker (0.1 Kb in the ladder well). Authors were unable to provide a full-length image for this gel.

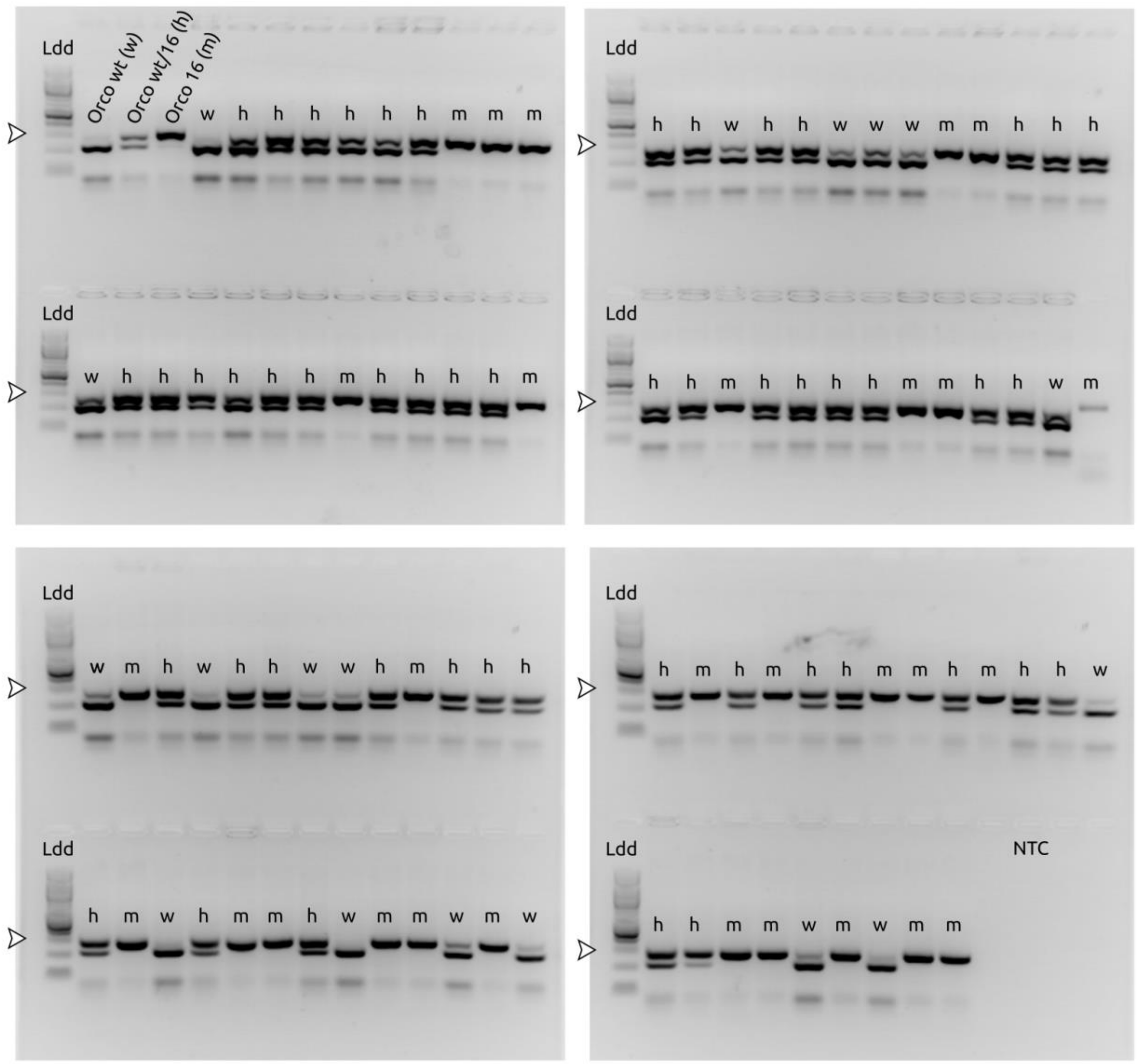

Supplementary Fig. S8 Genotyping by Cas9-cleavage assay. Siblings carrying a -16 bp deletion at *ChomOrco* were selected and inbreed at G_2. Their offspring were genotyped by the Cas9-cleavage assay method, and homozygous mutant flies let inbreed to establish the knockout strain used in this study. Abbreviations used: Ldd = 100 bp DNA Ladder (NEB), white arrows point to the 300 bp band; NTC = no template control; w = wildtype genotype ($Orco^{wt/wt}$); h = heterozygous genotype ($Orco^{16/wt}$); m = homozygous mutant genotype ($Orco^{16/16}$). Amplifications were conducted using the primers genOrco-F1 along with genOrco-R1 (Supplementary Table S4).

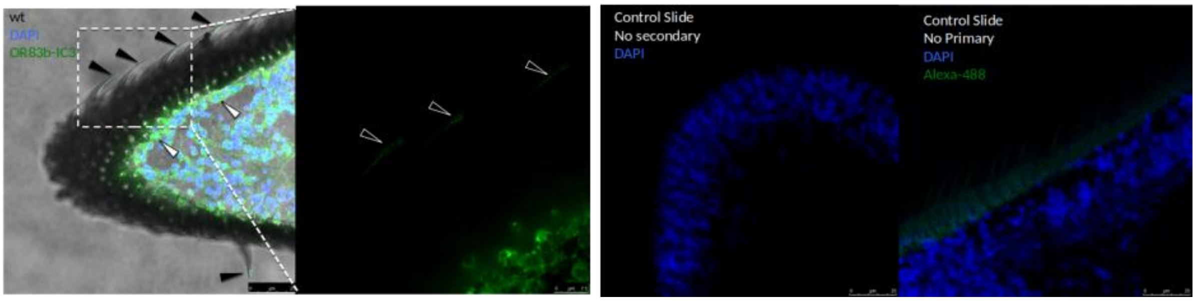

Supplementary Fig. S9 Control slides for immunostaining of *ChomOrco*. Wildtype (wt; *leftmost*) samples showed that *ChomOrco* protein is found in the cell body (white arrows) and dendrites (black arrows) of olfactory sensory neurons (OSNs). Control slides (*rightmost*) showed no specific labeling, but some nonspecific background in the absence of primary OR83b-IC3 antibody (same gain/exposition settings were used for these images and samples shown in Fig. 3F).

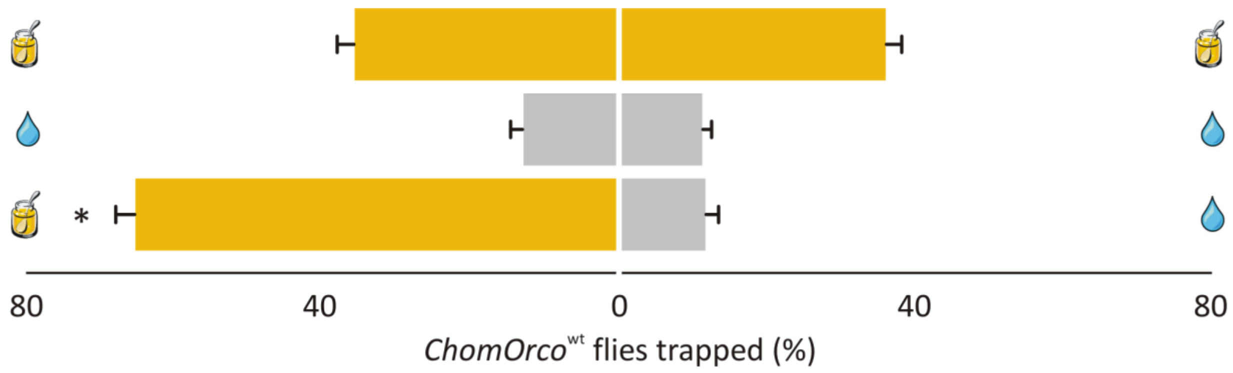

Supplementary Fig. S10 Response of wt screwworms (*ChomOrco*^{wt}) to honey (honey jar) or glycerol (drop) in two-choice trap assays. Screwworm adults distribute evenly between traps when both contain honey (Student's t-test; $p = 0.42$, $n = 11$), and show little or no response to glycerol ($n = 9$), indicating that they don't display preferences to any cage sides in our setup. However, flies show a strong preference for honey in opposition to glycerol ($p < 0.001$, $n = 12$). Preference Index was calculated as: PI = (n_{trap} / n_{total}) * 100, where n_{trap} is the number of flies captured in a given trap, and n_{total} the total of flies in the test cage.

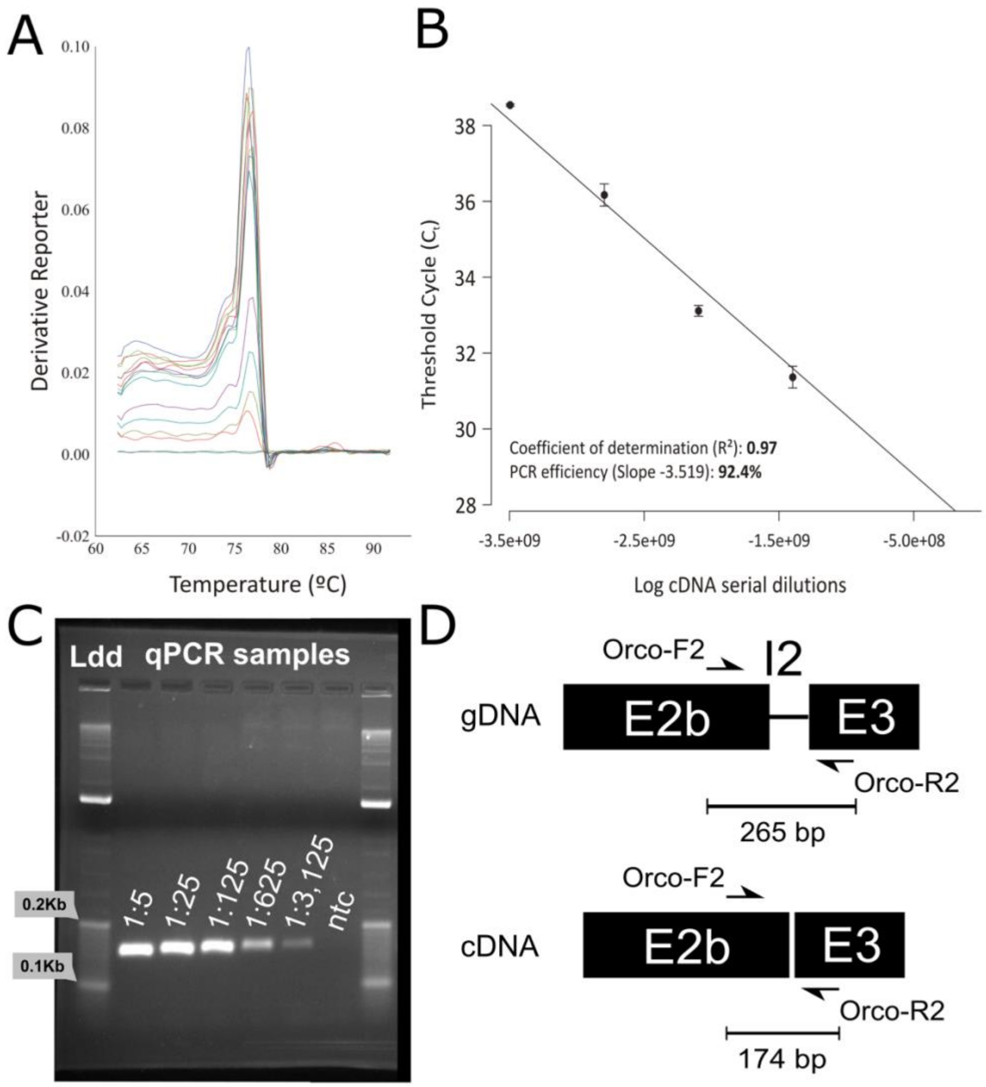

Supplementary Fig. S11 Quantitative PCR (qPCR) efficiency analysis. The amplification efficiency of the Orco-F2 and Orco-R2 primers (Supplementary Table S4) for qPCR experiments was evaluated by Standard Curve analysis using a 5-fold serial dilution of cDNA (leftmost bands in C, dilutions from 1:5 to 1:3,125). (**A**) Dissociation curve indicating single specific amplification of *ChomOrco* transcript. (**B**) The Threshold Cycle (C_T) results are presented with the respective standard deviations of the means (n = 3). Efficiency index was calculated as: $EI = -1 + 10^{-1/slope}$. (**C**) Amplifications and non-template controls (NTC), were resolved in a 2% agarose gel electrophoresis, confirming results obtained by dissociation curve analysis. (**D**) Amplification scheme of the intron-spanning strategy used for RT-PCRs, showing expected sizes for genomic DNA (gDNA) and complementary DNA (cDNA) amplifications of *ChomOrco* targeted sequences.

Supplementary Table S1 Sequence divergence between *ChomOrco* and other dipteran's orthologues. Uncorrected evolutionary distances (*p*-distance) based on nucleotide (nt), and amino-acid (aa) sequences were estimated using pairwise comparisons with pairwise site-deletions in MEGA7 [1]. Results are shown as Avg ± SE.

Sequence	*ChomOrco* Vs.	*p-distance*
nt	Diptera	0.21 ± 0.006
	Calyptratae	0.13 ± 0.004
	Calliphoridae	0.08 ± 0.004
	DmelOrco	0.27 ± 0.010
	CmegOrco	0.08 ± 0.007
aa	Diptera	0.08 ± 0.006
	Calyptratae	0.03 ± 0.004
	Calliphoridae	0.02 ± 0.005
	DmelOrco	0.09 ± 0.013
	CmegOrco	0.02 ± 0.005

Supplementary Table S2 Mutational inheritance of *Orco* mutant alleles. Injected screwworm males (putative founders) were genotyped using our non-lethal DNA extraction method and T7EN1 cleavage assay. Ten confirmed heterozygous flies (*Orco*$^{wt/-}$) were randomly selected and individually backcrossed to wt screwworm females. For each crossing, another 8 males (on average) were randomly sampled and genotyped as before. Transmission efficiency was found to be 90% (9 out of 10 crosses produced heterozygous offspring), while inheritance success (germline transmission) ranged from 14.3% to 88.9%. In total, 41% (*n* = 38 / 81) of the genotyped flies were found to be heterozygous.

Crossing ID	Orco $^{wt/-}$	*n*(G_1)	Inheritance
A	3	8	37,5%
B	8	9	88,9%
D	2	14	14,3%
E	1	6	16,7%
G	3	4	75,0%
H	5	8	62,5%
L	2	6	33,3%
N	4	5	80,0%
O	0	8	00,0%
Q	10	13	76,9%

Supplementary Table S3 Specification of species and *Orco* sequences used in the evolutionary analyses.

Species	Abbreviation	Orco gene	GenBank Accession	Taxonomy
Aedes aegypti	Aaeg	*AaegOrco*	NM_001358471	Diptera: Culicidae
Anopheles gambiae	Agam	*AgamOrco*	Q7QCC7	Diptera: Culicidae
Drosophila melanogaster	Dmel	*DmelOrco*	AY567998	Acalyptratae: Drosophilidae
Drosophila pseudoobscura	Dpse	*DpseOrco*	XM_001359327	Acalyptratae: Drosophilidae
Drosophila ananassae	Dana	*DanaOrco*	XP_001953343	Acalyptratae: Drosophilidae
Drosophila yakuba	Dyak	*DyakOrco*	XM_002096017	Acalyptratae: Drosophilidae
Drosophila suzukii	Dsuz	*DsuzOrco*	NM_001328601	Acalyptratae: Drosophilidae
Ceratitis capitata	Ccap	*CcapOrco*	XM_012300753	Acalyptratae: Tephritidae
Bactrocera cucurbitae	Bcuc	*BcucOrco*	HM745934	Acalyptratae: Tephritidae
Bactrocera dorsalis	Bdor	*BdorOrco*	EU621792	Acalyptratae: Tephritidae
Glossina morsitans	Gmor	*GmorOrco*	ND [1]	Calyptratae: Glossinidae
Musca domestica	Mdom	*MdomOrco*	JQ365179	Calyptratae: Muscidae
Stomoxys calcitrans	Scal	*ScalOrco*	EU622914	Calyptratae: Muscidae
Haematobia irritans	Hirr	*HirrOrco*	EU622915	Calyptratae: Muscidae
Lucilia sericata	Lser	*LserOrco*	HQ315862	Calyptratae: Calliphoridae
Lucilia cuprina	Lcup	*LcupOrco*	XM_023445888	Calyptratae: Calliphoridae
Calliphora stygia	Csty	*CstyOrco*	KJ702047	Calyptratae: Calliphoridae
Aldrichina grahami	Agra	*AgraOrco*	HQ190955	Calyptratae: Calliphoridae
Chrysomya ruffifacies	Cruf	*CrufOrco*	JQ365176	Calyptratae: Calliphoridae
Chrysomya megacephala	Cmeg	*CmegOrco*	HQ315861	Calyptratae: Calliphoridae
Cochliomyia macellaria	Cmac	*CmacOrco*	MT226797 [2]	Calyptratae: Calliphoridae
Cochliomyia hominivorax	Chom	*ChomOrco*	MT226798 [2]	Calyptratae: Calliphoridae

1. Retrieved from Obiero et al. [6]
2. Described in the present study.

Supplementary Table S4 Specifications for all the primers used in this study.

oligo	target	aim	dir.sequence (5' - 3')	length (nt)	tm (°C)	amplicon (bp)	off-targets [d]	mitSpecScore [d]
genOrco-F1	Orco E1 [a]	genotyping; long-PCR	+ GGT TAT GTC ATG TCG TTA CAG AAG	24	57	280	-	-
genOrco-R1		genotyping; HRMob	- GTT CTT TTG ATT GAC CGC TAG AT	23	57		-	-
delOrco-F1		genotyping; HRMob	+ ACT TGT CAA CAT GGC CCT GAA	21	60	120 (with delOrco-R1)	-	-
genOrco-F2	Orco E2b [a]	genotyping	+ GGA TAA CCA TCA CTT TCT TTG	21	52	360	-	-
genOrco-R2		genotyping; long-PCR	- TGG CTG ACA ATG AAC GAA AG	20	56		-	-
Orco-F1	Orco E2b	RACE; long-PCR	+ GTT CTT GGT TAA TAT TCG CCT G	22	55	660	-	-
Orco-R1	Orco E5	RACE; long-PCR; RT-PCR	- GGC ATT GTT GAC AAA CGA TTT G	22	57		-	-
Orco-F2	Orco E2b	qPCR; long-PCR	+ CAA CTA CAA CAT TTG AAG GG	20	55	174	-	-
Orco-R2	Orco E3	qPCR; RACE	- AGA TGC CCG ACA TAT CCA GG	20	59		-	-
Orco-F3	Orco E3	RT-PCR	+ CTGCTCTCTTTCGTTCWTTGTCAGCC	26	62	558 (with Orco-R1)	-	-
Orco-R3	Orco E4	long-PCR	- TTT GGT TGC CTG ATA CGC CA	20	60	-		
Gapdh-F1	*GAPDH* [b]	qPCR; RT-PCR	+ GTC AGT GAC ACC CAC TCC TC	20	59	128	-	-
Gapdh-R1			- TTG ATC AAG TCG ATG ACA CG	20	55		-	-
sgR-Orco-E1	Orco E1 [c]	CRISPR/Cas9	+ GAT GAA GTA AAT GAA TTG TC **CGG**	20	-	100 bp (with sgR-Uni-R)	0-0-1-3-37	93
sgR-Orco-E2b	Orco E2b [c]		- GAG TAA ATA GTA ACC TTG AA **AGG**	20	-	100 bp (with sgR-Uni-R)	0-1-0-1-16	71

[a] These genotyping primers were also used for Illumina library preparation by including Multiplexing Read 1 (5' – CAC TCT TTC CCT ACA CGA CGC TCT TCC GAT CT – 3') and Read 2 (5' – GTG ACT GGA GTT CAG ACG TGT GCT CTT CCG ATC T – 3') sequences at the 5'-end of the forward and reverse genotyping primers, respectively.

[B] These primers were previously evaluated by Cardoso et al. [7], and have been widely accepted for functional studies in blowflies due to their consistent results (e.g., Ct values) within and among different species of blowflies and their developmental stages.

[c] sgRNAs used in this study, PAM motif in red. Syntheses were performed as described by Bassett and Liu [8], using the primers sgR- Specific-T7-FWD (5'- GAA ATT AAT ACG ACT CAC TAT A(G) [specific sgRNA sequence without PAM] TTG GGT TTT AGA GCT AGA AAT AGC -3') and sgR-Universal-REV (5'- AAA AGC ACC GAC TCG GTG CCA CTT TTT CAA GTT GAT AAC GGA CTA GCC TTA TTT TAA CTT GCT ATT TCT AGC TCT AAA AC -3');

[d] sgRNAs were designed by using the standalone version of CRISPOR [9]. Potential off-targets were evaluated in the context of *C. hominivorax* draft assembly [10]. We considered sgRNAs: (1) targeting *ChomOrco* exons 1 and 2b, aiming the early disruption of the *Orco* gene in order to avoid truncated protein activity; (2) with the smaller number of potential off-targets, with preferentially ≥ 3 mismatches between the sgRNA and non-specific genome sequences; (3) with the maximum amount of mismatches present on the 5 first bases of the sgRNA directly upstream to PAM motif, which constitute the so called "*seed*" region; (4) starting with an "G" base, to improve T7 transcription initiation, and; (5) with an optimal genomic location for the design of genotyping primers.

Supplementary Methods

Classic RACE

For 3'-UTR isolation, a cDNA was synthesized with the RT-Q_t primer and used as template in a first RT-PCR with the primers RT-Q_0 and Orco-F1. The product was 20-fold diluted in 1X TE (10 mM Tris, 1 mM EDTA, pH 8.0) and used as template for a nested RT-PCR with the inner primers RT-Q_i and Orco-F2. The amplicon was TA cloned and sequenced. For 5'-UTR isolation, a cDNA was synthetized with the Orco-R1 primer, purified by organic extraction with Phenol: Chloroform: Isoamyl Alcohol (25:24:1, v/v, pH 6.7), precipitated with with sodium acetate (NaOAc; 3 M, pH 5.2) and absolute ethanol (EtOH) at -80 °C for 3 h, and resuspended in 1X TE. An artificial poly(A) tail was attached to the 5'-end of the cleaned cDNA with 30 U of terminal transferase (TdT, Invitrogen) and 200 µM of dATP. The reaction was denatured at 94 °C for 3 min, the tail synthesized at 37 °C for 30 min, and the TdT inactivated at 70 °C for 10 min, followed by a second EtOH precipitation. The tailed-cDNA was used as a template in the first RT-PCR with the primers RT-Q_t, RT-Q_0, and Orco-R1. Amplifications were conducted as before after an initial round of 95 °C for 5 min, 48 °C for 2 min, and 72 °C for 15 min. The product was diluted as before and used as a template for a nested RT-PCR with the inner primers RT-Q_i and Orco-R2. The resulting amplicon was cloned and sequenced bidirectionally. Overlapping fragments were assembled with CAP3 [11], coding sequences (CDS) were identified by (DELTA)-BLASTp analysis and annotated using the ExPASy translate tool [5].

Long-PCR Product Sequencing

Total DNA was extracted from frozen adult flies using the DNeasy Blood and Tissue Kit (Qiagen). Long-PCRs were performed in a 50 µl reaction, containing 0.2 µM of each forward and reverse primers (see Supplementary Table S4), 20 µM of dNTPs, 1X Reaction Buffer B, and 1 U of Elongase enzyme mix (Invitrogen). Amplification conditions consisted of an initial denaturation at 94 °C for 60 sec, 40 cycles of [94 °C for 30 sec, 50 °C for 30 sec and 68 °C for 8 min], followed by a final elongation at 68 °C for 16 min. Long amplicons were normalized to 10 ng/µl in 1X TE buffer for a final volume of 100 µl, and sheared by sonication in a Bioruptor plus UCD-300 (Diagenode) through 2 cycles of 15 sec "ON" and 90 sec "OFF", in a 4 °C water bath. Fragments were purified

with the PureLink PCR purification kit (Invitrogen) using the selective Buffer B3. Purified products (500 ng) were repaired in a reaction containing 1X T4 DNA polymerase Buffer, 1 mM dNTPs mix, 0.2 mg/ml of BSA and 15 U of T4 DNA polymerase (Invitrogen) for a final volume of 12 µl. Repair reactions were incubated at 12 °C for 15 min followed by the addition of 10 U of Klenow fragment (Thermo Scientific) and a second incubation at 37 °C for 30 min and 72 °C for 20 min. Blunt-end fragments were phosphorylated in a 100 µl reaction containing 1X T4 DNA ligase buffer supplemented with 1 mM of ATP and 25 U of T4 Kinase (Invitrogen). Phosphorylation was performed at 37 °C for 30 min and stopped at 75 °C for 20 min. Ready-to-clone fragments were precipitated with sodium acetate (3 M, pH 5.2) and absolute EtOH at -80 °C overnight, washed once with 70% EtOH, and resuspended in 1X TE. Sub-libraries were constructed by incubating 100 ng of each prepared fragments and linearized pBlueScript II SK (+) vector (Agilent) in a 20 µl reaction containing 1X T4 Rapid Ligation buffer and 3 U of T4 DNA ligase (Promega). Ligations were performed overnight at 16 °C and transformations were made overnight at 37 °C in DH10β competent cells. Sub-products were investigated via PCR, and clones hosting inserts around 900 bp were sequenced.

Intrapuparial Development

Screwworm pupae were collected three, six and eight days after pupation and fixed in Carnoy solution (EtOH: Acetic Acid: Chloroform, 6:3:1 (v/v)) for 48 h. Samples were then transferred to 5% formic acid for 48 h and transferred to 70% EtOH until imaging. Specimens were dissected with fine forceps and photographed with a Nikon P-FLAP 2 stereomicroscope system.

Electron Microscopy

Three-day-old adult females were freshly collected, fixed in absolute EtOH, and dehydrated through a graded EtOH series substituted with isoamyl acetate. Specimens were dried on a Denton DCP-1 Critical Point Drying apparatus (Denton Vaccum Inc.), mounted on aluminum stubs and sputter-coated with gold palladium alloy in a Hammer VI-A (Anatech) system. Specimens were imaged in a Zeiss EVO 40 scanning electron microscope (Carl Zeiss AG) at the Electron and Confocal Microscopy Laboratory, Smithsonian Tropical Research Institute (Earl S. Tupper Center).

Illumina Sequencing

Genomic DNA from G_0 flies transiently expressing the ZsGreen marker (Supplementary Fig. S2) were used as template in PCR amplifications spanning the Cas9-targeted sites. Amplicons were gel extracted using the QIAquick Gel Extraction Kit (Qiagen) and used as templates in a second PCR to incorporate Illumina adapters and barcodes (Supplementary Table S4). Pooled libraries were sequenced on an Illumina MiSeq (250 bp, paired-end run). Resulting raw reads were cleaned using Trimmomatic [12], connected with COPE [13], and aligned to the wt reference sequence using BWA-MEM [14]. Alignments were inspected for the presence of indels using CRISPResso [15] and CrispRVariants [4] pipelines.

Non-lethal DNA isolation

Genomic DNA extractions were carried out from a single midleg of adult flies (Supplementary Fig. S4). Dissected legs were frozen at -80 °C for at least 20 min and homogenized in 50 µl of fresh prepared Proteinase K solution (0.5 mg/mL Proteinase K, 10 mM Tris-HCL, 1 mM EDTA, and 25 mM NaCL in ddH_2O). The mixtures were incubated at 37 °C for 60 min followed by enzyme inactivation at 95 °C for 10 min. Extractions were kept at -20 °C until genotyping assays.

Trap's crafting

Traps were handmade of plastic soft drink bottles and contained two parts (Fig. 4B). A collection chamber was made of the upper cone-shaped funnel part of the bottle (60 mm height and 70 mm Ø), and a bait chamber made with the bottom part of the bottle (30 mm height). Two entries (35 mm apart from each other) were made in the collection chamber with an "*X*" shape cut. The resulting triangular portions were folded-in and used to support a 1 mL pipette tip cuted at the proximal end, which allows flies to enter the traps but prevents them from escaping. Baits were placed in the center of plastic dishes in the inner bottom of the bait chamber (35 mm Ø).

Two-choice assay movie

Supplementary Movie S1 shows that screwworm *Orco* mutants display Impaired host-seeking behavior (Related to Fig. 4). The movie displays two-choice trap assays showing side-by-side the response of 6-to-9-days-old female wt flies (*leftmost*) and *ChomOrco*^{16} mutants (*rightmost*) to

oviposition media. *Orco* mutants display a lack of decision-making and impaired flight orientation towards the stimuli source, while wt female flies are strongly attracted to the odors released from the oviposition device. The assay was recorded using a Canon EOS Rebel T3i under a monochrome mode. For illustration purposes, the movie plays only the first 5 min of each assay at 16X speed.

Supplementary References

1. Kumar, S., Stecher, G. & Tamura, K. MEGA7: Molecular Evolutionary Genetics Analysis Version 7.0 for Bigger Datasets. *Mol. Biol. Evol.* **33**, 1870–1874 (2016).
2. Tsirigos, K. D., Peters, C., Shu, N., Käll, L. & Elofsson, A. The TOPCONS web server for consensus prediction of membrane protein topology and signal peptides. *Nucleic Acids Res.* **43**, W401-407 (2015).
3. Junqueira, A. C. M. *et al.* Large-scale mitogenomics enables insights into Schizophora (Diptera) radiation and population diversity. *Sci. Rep.* **6**, 21762 (2016).
4. Lindsay, H. *et al.* CrispRVariants charts the mutation spectrum of genome engineering experiments. *Nat. Biotechnol.* **34**, 701–702 (2016).
5. Gasteiger, E. *et al.* ExPASy: The proteomics server for in-depth protein knowledge and analysis. *Nucleic Acids Res.* **31**, 3784–3788 (2003).
6. Obiero, G. F. O. *et al.* Odorant and Gustatory Receptors in the Tsetse Fly Glossina morsitans morsitans. *PLoS Negl. Trop. Dis.* **8**, e2663 (2014).
7. Cardoso, G. A., Matiolli, C. C., de Azeredo-Espin, A. M. L. & Torres, T. T. Selection and validation of reference genes for functional studies in the Calliphoridae family. *J. Insect Sci. Online* **14**, 2 (2014).
8. Bassett, A. & Liu, J.-L. CRISPR/Cas9 mediated genome engineering in Drosophila. *Methods San Diego Calif* **69**, 128–136 (2014).
9. Concordet, J.-P. & Haeussler, M. CRISPOR: intuitive guide selection for CRISPR/Cas9 genome editing experiments and screens. *Nucleic Acids Res.* **46**, W242–W245 (2018).
10. Scott, M. J. *et al.* Genomic analyses of a livestock pest, the New World screwworm, find potential targets for genetic control programs. *Commun. Biol.* **3**, 1–14 (2020).
11. Huang, X. & Madan, A. CAP3: A DNA sequence assembly program. *Genome Res.* **9**, 868–877 (1999).
12. Bolger, A. M., Lohse, M. & Usadel, B. Trimmomatic: a flexible trimmer for Illumina sequence data. *Bioinforma. Oxf. Engl.* **30**, 2114–2120 (2014).
13. Liu, B. *et al.* COPE: an accurate k-mer-based pair-end reads connection tool to facilitate genome assembly. *Bioinformatics* **28**, 2870–2874 (2012).
14. Li, H. Aligning sequence reads, clone sequences and assembly contigs with BWA-MEM. *ArXiv Prepr. ArXiv13033997* (2013).

15. Pinello, L. *et al.* Analyzing CRISPR genome-editing experiments with CRISPResso. *Nat. Biotechnol.* **34**, 695–697 (2016).
